# Maturation of White Adipose Tissue Function in C57BL/6j Mice From Weaning to Young Adulthood

**DOI:** 10.3389/fphys.2019.00836

**Published:** 2019-07-09

**Authors:** Andrea Kodde, Eefje Engels, Annemarie Oosting, Kees van Limpt, Eline M. van der Beek, Jaap Keijer

**Affiliations:** ^1^Danone Nutricia Research, Utrecht, Netherlands; ^2^Department of Pediatrics, University Medical Center Groningen – University of Groningen, Groningen, Netherlands; ^3^Human and Animal Physiology, Wageningen University, Wageningen, Netherlands

**Keywords:** white adipose tissue, mitochondria, browning, maturation, uncoupling protein, oxidative phosphorylation, obesity

## Abstract

White adipose tissue (WAT) distribution and WAT mitochondrial function contribute to total body metabolic health throughout life. Nutritional interventions starting in the postweaning period may impact later life WAT health and function. We therefore assessed changes in mitochondrial density and function markers in WAT depots of young mice. Inguinal (ING), epididymal (EPI) and retroperitoneal (RP) WAT of 21, 42 and 98 days old C57BL/6j mice was collected. Mitochondrial density [citrate synthase (CS), mtDNA] and function [subunits of oxidative phosphorylation complexes (OXPHOS)] markers were analyzed, together with gene expression of browning markers (*Ucp1, Cidea*). mRNA of ING WAT of 21 and 98 old mice was sequenced to further investigate functional changes of the mitochondria and alterations in cell populations. CS levels decreased significantly over time in all depots. ING showed most pronounced changes, including significantly decreased levels of OXPHOS complex I, II, and III subunits and gene expression of *Ucp1* (PN21-42 and PN42-98) and *Cidea* (PN42-98). White adipocyte markers were higher at PN98 in ING WAT. Analyses of RNA sequence data showed that the mitochondrial functional profile changed over time from “growth-supporting” mitochondria focused on ATP production (and dissipation), to more steady-state mitochondria with more diverse functions and higher biosynthesis. Mitochondrial density and energy metabolism markers declined in all three depots over time after weaning. This was most pronounced in ING WAT and associated with reduced browning markers, increased whitening and an altered metabolism. In particular the PN21-42 period may provide a time window to study mitochondrial adaptation and effects of nutritional exposures relevant for later life metabolic health.

## Introduction

Obesity prevalence is high in adults and considerably increased nowadays in children and adolescents (Ng et al., 2014). Childhood obesity increases the risk for early onset metabolic diseases, like type 2 diabetes mellitus (T2D) and cardiovascular disease (Reilly and Kelly, 2011). An important link between obesity and metabolic diseases is the metabolic function of the white adipose tissue (WAT), i.e., WAT health (Hammarstedt et al., 2012).

Mitochondrial density in adulthood appears to be strongly correlated to WAT health as shown by a reduced WAT mitochondrial density in obesity (Wilson-Fritch et al., 2004) and T2D (Choo et al., 2006). Nutrition may regulate WAT function as feeding a high fat diet reduced WAT mitochondrial density (Sutherland et al., 2008), a process that is already initiated after 5 days of western style diet (WSD) (Derous et al., 2015), while caloric restriction and diets enriched in poly-unsaturated fatty acids increased WAT mitochondrial density, oxidative capacity and biogenesis (Flachs et al., 2005; Nisoli et al., 2005). Dependent on their location in the body, WAT depots differ in their impact on metabolic health (Yang et al., 2008; Bjorndal et al., 2011). Visceral WAT is located in the abdominal cavity and visceral WAT mass is inversely correlated to total body insulin sensitivity and as such considered a risk factor for development of the metabolic syndrome (Pouliot et al., 1992; Ross et al., 2002). In contrast, subcutaneous WAT is located directly under the skin and is shown to have a higher oxidative capacity compared to the visceral depots in mice (Schottl et al., 2015).

Experimental evidence suggests that growth and distribution of WAT as well as mitochondrial density of WAT depots can be programmed by early life environmental factors. For example, maternal obesity, over-nutrition or undernutrition during pregnancy can all drive increased visceral adiposity and an adapted mitochondrial density in rodent offspring (Bruce et al., 2009; Jousse et al., 2014; Claycombe et al., 2016; Lecoutre et al., 2016). In addition, mild caloric restriction or a high fat diet exposure in the lactation period also programmed adult adiposity and metabolic health of pups (Mitra et al., 2009; Palou et al., 2010). Those studies show that suboptimal nutrient conditions in early life can have long-term metabolic consequences. Programming of the oxidative and storage capacity of WAT, which develops from the third trimester of gestation until adolescence (Spalding et al., 2008), may be an underlying mechanism.

The postnatal development of WAT includes differentiation from progenitor cells to fully developed adipocytes containing lipid droplets, a process starting before birth in subcutaneous depots and after birth in visceral depots (Han et al., 2011; Wang et al., 2013). After weaning WAT depots continue to grow and adipocytes increase in size (hypertrophy) and number (hyperplasia) in a depot specific manner (DiGirolamo et al., 1998). Furthermore, WAT depots develop postnatally from a white phenotype at PN10 to a brown phenotype at PN20 where the majority of the adipocytes are multilocular and express UCP1, after which these cells disappear again and are replaced by unilocular adipocytes which only express UCP1 upon cold-induction (Xue et al., 2007; Lasar et al., 2013; Birnbacher et al., 2018).

Many nutritional intervention studies, including postnatal programming studies, i.e., studies with a nutritional intervention in early life with the aim to improve adult metabolic health (Baars et al., 2016; Kodde et al., 2017; Bouwman et al., 2018; Fernandez-Calleja et al., 2018), take the postweaning period (around PN21) as starting point. Comprehensive and extended comparison of postweaning changes of markers for mitochondrial function and WAT browning in different WAT depots is crucial for the interpretation of those studies. Therefore, we here investigate early life changes in markers for mitochondrial density, function and browning in the developing WAT depots with the aim to substantiate and extend, in terms of number of markers and WAT depots analyzed, available research. To this end we collected inguinal (ING), epididymal (EPI) and retroperitoneal (RP) WAT, of 21, 42, and 98 days old mice, housed under standardized experimental conditions (ambient temperature) and comprehensively measured markers of mitochondrial density, function and browning as well as the effect of a WSD on these markers. In addition, we newly examined changes within mitochondrial functional profile by analyzing transcription of all established mitochondrial proteins and categorize them to function.

## Materials and Methods

### Study Design

**FIGURE 1:**
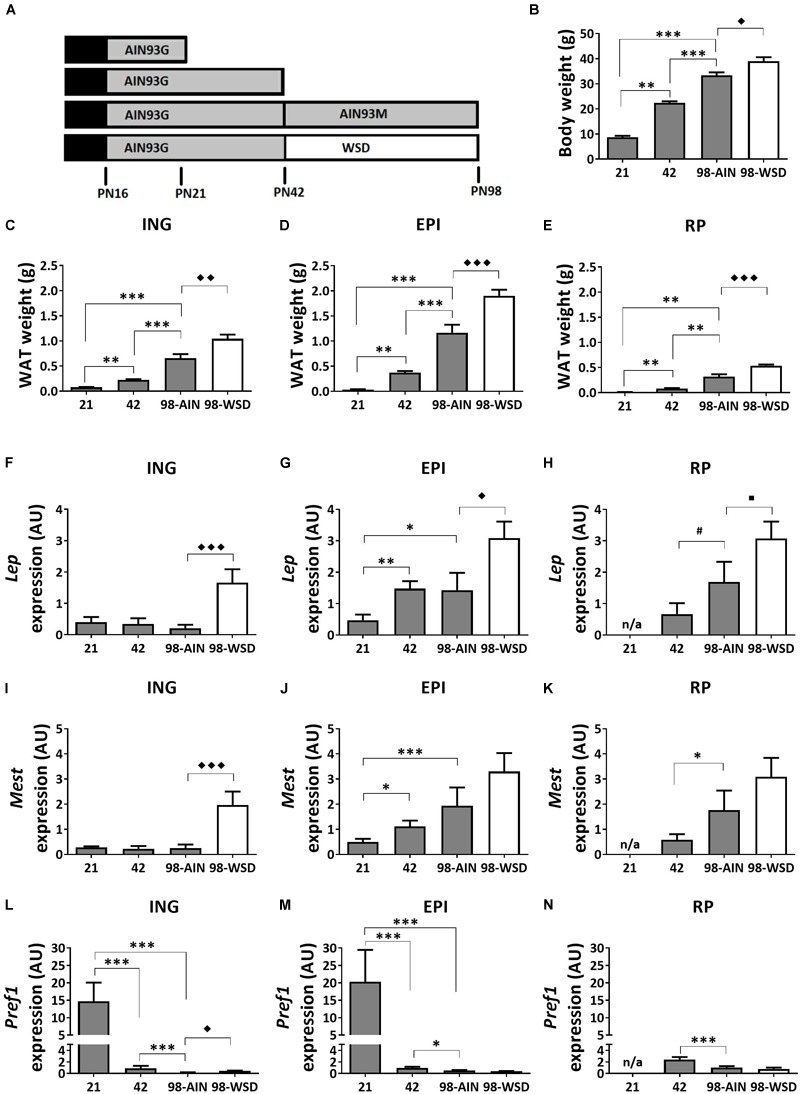
Study design, development of body weight, WAT weight and gene expression of adiposity markers between PN21 and PN98. **(A)** Study design with at PN16 start of weaning to full weaning at PN21; **(B)** body weight; weight of **(C)** ING WAT, **(D)** EPI WAT, **(E)** RP WAT; *Lep* expression in **(F)** ING WAT, **(G)** EPI WAT, **(H)** RP WAT; *Mest* expression in **(I)** ING WAT, **(J)** EPI WAT, **(K)** RP WAT; *Pref1* expression in **(L)** ING WAT, **(M)** EPI WAT, and **(N)** RP WAT. PN, postnatal day; AIN, AIN-93G diet from PN16-42 and AIN-93M from PN42-98 (gray); WSD, western style diet (white); n/a, data not available. Data expressed as mean + SEM. WAT weight: *n* = 8 for PN 21 and PN42, *n* = 11 for PN98; gene expression: *n* = 8 for PN 21 and PN42, *n* = 11 for PN98, no data available for RP WAT at PN21. Time and WSD effects were analyzed separately; time effect: ^∗^*p* < 0.05; ^∗∗^*p* < 0.01; ^∗∗∗^*p* < 0.001; ^#^0.05 < *p* < 0.1; WSD effect: ^◆^*p* < 0.05; ^◆◆^*p* < 0.01; ^◆◆◆^*p* < 0.001; WSD effect: ^■^0.05 < *p* < 0.1.

Mice were kept at the animal facility of Intravacc (Bilthoven, Netherlands) under a 12 h light – 12 h dark cycle (lights on at 06:00 h). Room temperature and humidity were kept at constant level (21 ± 2°C and 50 ± 5%, respectively). This housing temperature was chosen to adhere to the most common temperature used for animal experiments. C57BL/6jOlaHsd breeders were purchased from Harlan (Envigo since 2015, Horst, Netherlands), acclimatized for 2 weeks, time mated and fed a American Institute of Nutrition-93G synthetic diet (AIN93G) (Reeves et al., 1993) during breeding, pregnancy and lactation. Within 2 days after birth, litters were culled to four males and two females and randomly assigned to a dam. At postnatal day 21 (PN21) female mice were killed, while male mice were weaned, housed in littermate-pairs and continued on AIN93G until PN42. From PN42 until sacrifice at PN98 mice were fed AIN93M (Reeves et al., 1993) or WSD (containing 39 en% fat; diet composition in Table 1). Food and water were available *ad libitum* during the entire experimental period. A very limited amount of food was supplied the night before dissection to ensure that the animals were in a fasted state (approximately 8 h). Body weight and food intake were measured twice a week. At different time points (PN21, PN42, and PN98) mice were sacrificed to assess the development of WAT depots and the effect of the WSD challenge, resulting in the following experimental groups (i) PN21 (*n* = 8), (ii) PN42 (*n* = 8), (iii) PN98-AIN (*n* = 11), and (iv) PN98-WSD (*n* = 11; Figure 1A). At dissection mice were anesthetized (isoflurane/N_2_O/O_2_), terminated by bleeding (eye extraction) and ING, EPI, and RP WAT were collected, weighted, snap frozen and stored at -80°C.

**Table 1:** Diet composition.

Diet		AIN93GG	AIN93M	WSD
**Carbohydrates**	(g/kg)	629.5	720,7	499.5
Dextrose	(g/kg)	–	50	150
Sucrose	(g/kg)	100	100	–
Starch	(g/kg)	397.5	415.7	150
Maltodextrin	(g/kg)	132	155	199.5
**Proteins**	(g/kg)	203	141.8	203
Casein	(g/kg)	200	140	200
Cystine	(g/kg)	3.0	1.8	3.0
**Fat**	(g/kg)	70	40	200
Soy oil	(g/kg)	70	40	30
Lard	(g/kg)	–	–	169
Cholesterol	(g/kg)	–	–	1.0
Fiber (Cellulose)	(g/kg)	50	50	50
Vitamin and mineral mix	(g/kg)	47.5	47.5	47.5
**Energy %**				
Carbohydrates	(en%)	64	76	43
Proteins	(en%)	21	15	18
Fat	(en%)	16	9	39

### Sample Homogenization

The EPI, ING, and RP WAT depots were homogenized with a Cellcrusher (Cellcrusher, Cork, Ireland) and aliquots of the resulting homogenized powder where made to enable separate analyses. EPI and RP WAT at PN21 were too small to make aliquots and were therefore only used for RNA extractions and enzyme activity measurements, respectively. In addition, RP WAT of PN42 was too small to analyze mitochondrial DNA levels.

### Gene Expression

RNA of EPI, ING and RP WAT was isolated using Trizol (Thermo Fisher Scientific, Landsmeer, Netherlands) followed by purification with a RNeasy Mini Kit (Qiagen Benelux b.v., Zwijndrecht, Netherlands) including a DNase treatment with a RNase-free DNase Set (Qiagen Benelux b.v.) as previously described (Vanhoutvin et al., 2009). RNA quantity and chemical purity were assessed with the Nanodrop 2000 (Thermo Fisher Scienctific) and integrity with the Agilent 2100 Bioanalyzer (Agilent, Santa Clara, CA, United States). iScriptcDNA synthesis kit (Bio-Rad, Veenendaal, Netherlands) was used according to manufacturer instructions. 6.25 ng cDNA was used as input for each Q-PCR reaction. SYBR Select Master Mix (Life Technologies Europe, Bleiswijk, Netherlands) was used according to manufacturer instructions and qPCR was performed with a QuantStudio 6 Flex Real-Time PCR System (Life Technologies Europe). mRNA expression of cell death-inducing DNA fragmentation factor, alpha subunit-like effector A (*Cidea*), Leptin (*Lep*), mesoderm specific transcript (*Mest*, also known as *Peg1*), delta-like 1 homolog (*Dlk1*, better known as *Pref1*, which will be used here) and uncoupling protein 1 (*Ucp1*) were analyzed relative to mean expression of two reference genes, hypoxanthine guanine phosphoribosyl transferase (*Hprt*) and zinc finger, AN1-type domain 6 (*Zfand6*). For a complete list of primers used see Table 2. Normalization of qPCR data was performed using the qbase^+^ (Biogazelle, Genth, Belgium) based on the method of relative normalization as described (Hellemans et al., 2007; Kodde et al., 2017). Reference genes *Hprt1* and *Zfand6* were selected based on transcriptome data and stability checked by Q-PCR analyses. Primers for *Ucp1* were purchased from Biorad, all other primers from Biolegio (Biolegio, Nijmegen, Netherlands).

**Table 2:** Primer sequences.

Gene name	NCBI reference number	Forward primer (5′ – 3′)	Reverse primer (5′ – 3′)
***Genes of interest:***		
*Cidea*	NM_007702.2	aggccgtgttaaggaatctg	cccagtactcggagcatgta
*Lep*	NM_008493_3	aggatgacaccaaaaccctcat	agtccaagccagtgaccctct
*Mest*	NM_008590.1	tcagtgacaagccgagacca	gttgattctgcggttctggag
*Pref1*	NM_010052.5	tgcgaggctgacaatgtctg	atgcactgccatggttcctt
*Ucp1*		assay ID qMmuCID0005832	
**Reference genes:**		
*Hprt*	NM_013556.2	ggacctctcgaagtgttggat	ccaacaacaaacttgtctggaa
*Zfand6*	NM_022985.6	tgggacttactgggtttgaa	ttctcagcagcatcagcttt
***DNA primers:***		
*mt-Nd1*	NC_005089.1	accaatacgccctttaacaac	aatgggtgtggtattggtagg
*Lpl*	NM_008509	tcctgatgacgctgattttg	atgtcaacatgccctactgg

### Enzyme Activity

Citrate synthase (CS) and hydroxyacyl-Coenzyme A dehydrogenase (HADH) activities were measured as described (Shepherd and Garland, 1969; Kodde et al., 2017).

### OXPHOS and UCP1 Protein Levels

Protein levels of five subunits (NDUFB8, SDHB, UQCRC2, MTCOI, ATP5A1) representing the five oxidative phosphorylation (OXPHOS) complexes (I–V, respectively) were measured with western blot (Meex et al., 2010; Kodde et al., 2017). Briefly, 15 μg of total protein per sample was used for SDS-PAGE, transfer to a PVDF membrane was done using the TransBlot Turbo (Biorad). Blots were blocked with 5% Protifar (Nutricia, Zoetermeer, Netherlands), incubated with Mito-Profile Total OXPHOS rodent western blot antibody cocktail (Abcam, Cambridge, United Kingdom) as primary antibody and ECL anti mouse IgG (Thermo Fisher Scienctific) as a secondary antibody. OXPHOS protein levels, detected with Supersignal West Dura (Thermo Fisher Sceintific), were related to total protein levels as analyzed by Coomassie brilliant blue staining. Protein levels were detected with the Chemidoc XRS and analyzed by Quantity One (Biorad). The same procedure was used for detection of UCP1 protein levels, except that 5% BSA was used as blocking agent, rabbit anti mouse UCP1 (Abcam, Cambridge, United Kingdom) was used as primary antibody, goat anti rabbit IgG (Santa Cruz, Dallas, United States) as secondary antibody and Supersignal West Femto (Thermo Fisher Scientific) was used for signal detection. A representative Western blot stained for the OXPHOS and for UCP1 proteins and the corresponding Coomassie brilliant blue stained blots are shown in Supplementary Figures 3, 4, respectively.

### Mitochondrial DNA Density

Mitochondrial copy number was assessed by the ratio (ΔCt) between nuclear DNA (abundance of lipoprotein lipase (*Lpl*) DNA) and mitochondrial DNA (abundance of mitochondrial gene NADH dehydrogenase 1 (*mt-Nd1*) DNA) (Kaaman et al., 2007). Briefly, total DNA was isolated with the QIAamp DNA micro kit (Qiagen Benelux), following instructions of the manufacturer. DNA quantity was determined with Quant-iT Picogreen dsDNA assay kit (Thermo Fisher Scientific). 10 ng input DNA was used for each qPCR reaction. Primers sequences are shown in Table 2.

### mRNA Sequencing

RNA samples of ING depots from PN21 and PN98 (*n* = 4 per time point) were used for mRNA sequencing analysis (NXT-Dx, Gent, Belgium). After RNA quantification (Rediplate 96 Ribogreen RNA Quantitation kit, Life Technologies), RNA libraries were prepared (NEBNext Ultra Directional RNA library Prep Kit for Illumina, New England Biolabs INC., Ipswich, United Kingdom), intact poly(A)+ RNA was isolated (NEBNext Poly(A) mRNA Magnetic isolation Module, New England Biolabs INC., Ipswich, United Kingdom) and cDNA libraries were sequenced (paired end, 100 base pairs) on an Illumina HiSeq sequencer (Illumina Netherlands, Eindhoven, Netherlands). FASTQ sequences were generated using the Illumina Casava pipeline 1.8.2. Quality of the data was assessed using the Illumina Chasitity filter and quality of the reads using the FASTQC quality control tool version 0.10.0. Subsequently, data were mapped on the mouse reference genome mm10 using STAR Aligner 2.3.0 and analyzed with Cufflinks v2.1.1 on Gencode annotation v15. mRNA sequence data have been deposited at the NCBI Gene expression omnibus (GSE116313).

### Data Analyses mRNA Sequence Data

Data analysis included contrast analysis (R package Limma) between PN21 and PN98, omitting transcripts whit a FPKM (fragments per kilobase million) of zero in at least one of the samples and including transcripts with an average FPKM > 2 at PN21 or PN98. Principal Component Analysis (PCA) was performed to visualize the samples and results are reported in Supplementary Figure 1. Transcripts with a *p*-value below 0.05 were used for Ingenuity Pathway Analysis (Qiagen Bioinformatics, Aarhus, Denmark) and targeted analysis as described below.

The data set was examined for changes in brown and white (pre)adipocyte markers derived from Gesta et al. (2007) to get insight in the change in cell population in ING WAT between PN21 and 98.

To further explore changes in mitochondrial function, a list of genes encoding proteins with strong support of mitochondrial localization was derived from Mitocarta (Mouse MitoCarta 2.0, Broad institute), checked for their regulation in the data set, annotated with Nextprot (SIB Swiss institute for bioinformatics) and sorted per function category. When more than one transcript per gene was present in the data set, the transcript with the lowest *p*-value was used. Some of the genes are listed as non-mitochondrial (11 of 327) in the results table, because these are annotated in Nextprot having a ribosomal, extracellular matrix or cell membrane localization rather than a mitochondrial localization.

Next to this, a list of genes known to be involved in mitophagy was examined in the same data set, to get a better understanding of underlying mechanisms for WAT whitening.

### Statistical Analysis

SPSS 19.0 (SPSS Benelux, Gorinchem, Netherlands) was used for statistical analyses. Gaussian distribution was tested with Levene’s test for equality of error variances in all parameters. Differences over time (per depot) were analyzed using Univariate ANOVA. Depot differences over time were analyzed using a two-way ANOVA (Brown-Forsyte) with time and depot as factors. A *t*-test was used to analyze the effect of the WSD. Data that did not show a Gaussian distribution was analyzed by Kruskall-Wallis for time differences and Mann-Whitney for the adult diet effect. qPCR data is presented as mean relative expression (scaled to average expression) + SEM and all other data is displayed as mean + SEM. Differences were considered significant at *p* < 0.05 and tendency was reported when 0.05 < *p* < 0.1. Correlations were analyzed with Pearson’s test.

## Results

### Body Weight, WAT Weight and Markers of Adiposity

Body weight increased significantly between weaning (PN21) and young adulthood (PN98) (*p* < 0.001) (Figure 1B), as did the weight of ING, EPI and RP WAT (*p* < 0.01; Figure 1C–E). In accordance with increased WAT weight, gene expression levels of adiposity (*Lep*) and adipocyte expansion (*Mest*) markers increased over time in EPI WAT (*p* < 0.05 for *Lep* and *p* < 0.001 for *Mest*) and RP WAT (*p* = 0.06 for *Lep* and *p* < 0.05 for *Mest*), but not ING WAT (Figure 1F–K). *Mest* and *Lep* expression levels were increased in ING WAT upon WSD exposure (*p* < 0.001 for both parameters; Figure 1F,I). *Lep* expression levels were also moderately increased in EPI and RP WAT upon WSD (*p* < 0.05 for EPI and *p* = 0.08 for RP WAT; Figure 1G,H), whereas *Mest* expression levels were unaffected upon WSD in EPI and RP WAT (Figure 1J,K).

*Pref1* expression levels, a pre-adipocyte number marker, decreased in all depots over time (*p* < 0.001) and most pronounced from PN21 to 42 (Figure 1L–N). In contrast, *Pref1* expression levels were in EPI and RP WAT not affected by the WSD, but levels were slightly elevated in ING WAT (*p* < 0.01).

### Markers of Mitochondrial Density

**FIGURE 2:**
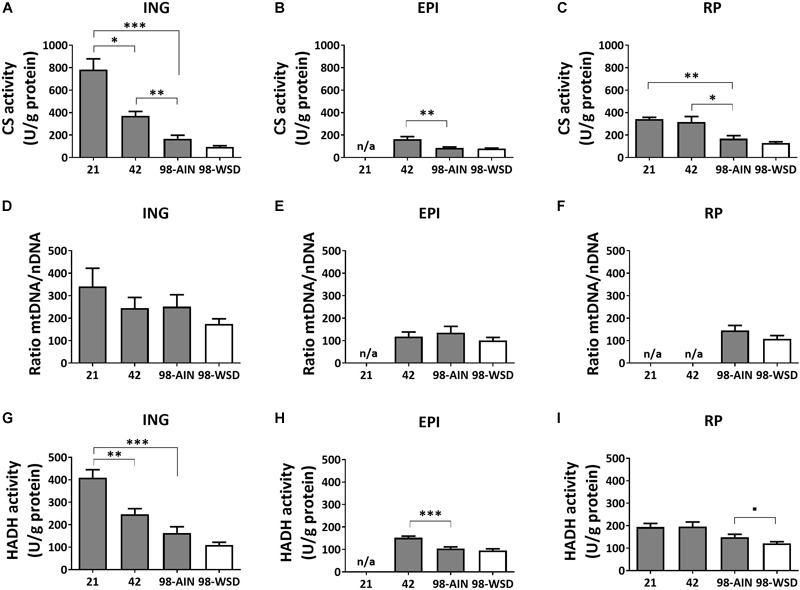
Changes in mitochondrial content markers, citrate synthase (CS), mtDNA and hydroxyacyl-Coenzyme A dehydrogenase (HADH), between PN21 and PN98. CS activity in **(A)** ING WAT, **(B)** EPI WAT, **(C)** RP WAT; mtDNA copy number in **(D)** ING WAT, **(E)** EPI WAT, **(F)** RP WAT; HADH activity in **(G)** ING WAT, **(H)** EPI WAT, and **(I)** RP WAT. PN, postnatal day; AIN, AIM93-M diet; WSD, western style diet. Data expressed as mean + SEM, *n* = 8 for PN 21 and PN42 and *n* = 11 for PN98, no data available (n/a) for EPI WAT at PN21 and for RP WAT at PN21 and PN42 for mtDNA copy number. Time and WSD effects were analyzed separately; time effect: ^∗^*p* < 0.05; ^∗∗^*p* < 0.01; ^∗∗∗^*p* < 0.001; WSD effect: ^■^0.05 < *p* < 0.1.

Mitochondrial density measured by citrate synthase (CS) levels, assayed as activity, decreased over time in all WAT depots (*p* < 0.01; Figure 2A–C). Between PN21 and PN42 CS levels decreased substantially (-56%) in ING WAT (*p* < 0.05). CS levels were not affected by the WSD in any depot (Figure 2A–C). Activity-based levels of hydroxyacyl-Coenzyme A dehydrogenase (HADH), a mitochondrial enzyme involved in β-oxidation, decreased over time in ING and EPI WAT (*p* < 0.001; Figure 2G,H), but not in RP WAT (Figure 2I). However, HADH activity tended to decrease in RP WAT upon WSD exposure (*p* = 0.07; Figure 2G–I). When mitochondrial density was measured as mtDNA copy number no significant decrease over time or upon WSD was found (Figure 2D–F).

### Markers of Mitochondrial Oxidative Capacity

**FIGURE 3:**
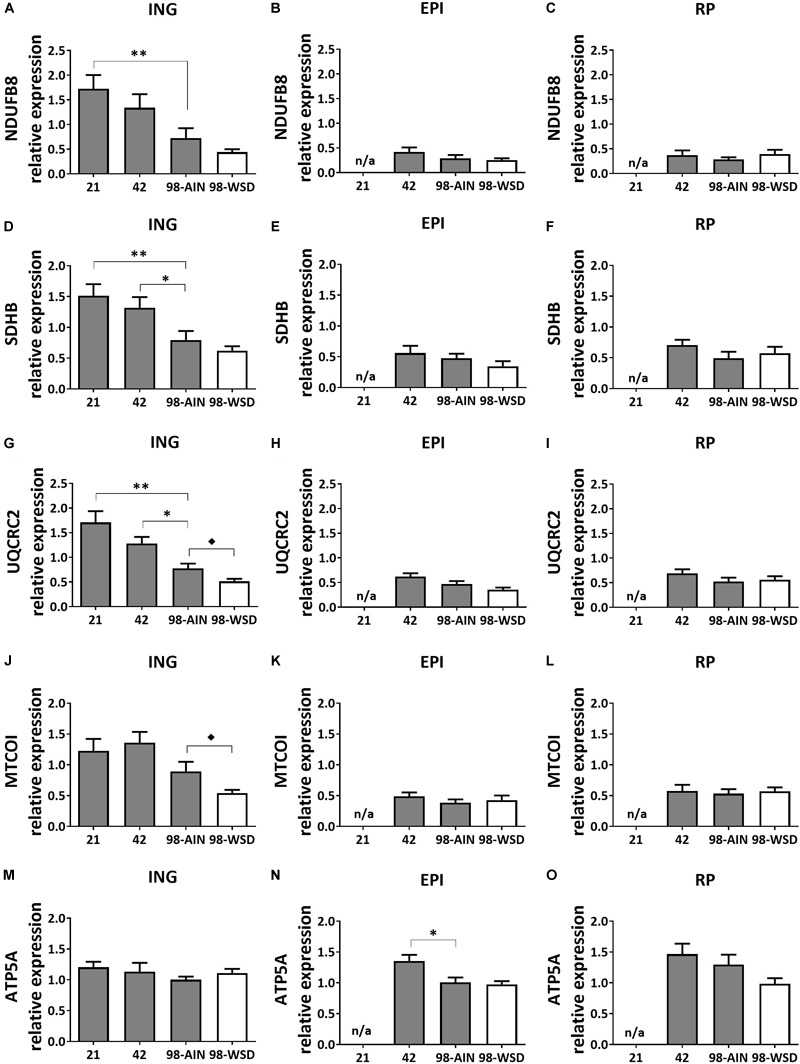
Changes in protein levels of subunits of oxidative phosphorylation complexes I-V between PN21 and PN98. NDUFB8 (Complex I) levels in **(A)** ING WAT, **(B)** EPI WAT, **(C)** RP WAT; SDHB (complex II) levels in **(D)** ING WAT, **(E)** EPI WAT, **(F)** RP WAT; UQCRC2 (complex III) levels in **(G)** ING WAT, **(H)** EPI WAT, **(I)** RP WAT; MTCOI (complex IV) levels in **(J)** ING WAT, **(K)** EPI WAT, **(L)** RP WAT; ATP5A (complex V) levels in **(M)** ING WAT, **(N)** EPI WAT, and **(O)** RP WAT. PN, postnatal day; AIN, AIM93-M diet; WSD, western style diet. Data expressed as mean expression levels corrected for total protein expression (Coomassie staining) + SEM, *n* = 8 for PN 21 and PN42 and *n* = 11 for PN98, no data available (n/a) for EPI and RP WAT at PN21. Time and WSD effects were analyzed separately; time effect: ^∗^*p* < 0.05; ^∗∗^*p* < 0.01; WSD effect: ^◆^*p* < 0.05.

Mitochondrial oxidative capacity, measured by protein levels of five subunits representing the five oxidative phosphorylation (OXPHOS) complexes, decreased in ING WAT over time for NDUFB8 (*p* < 0.01; Figure 3A), SDHB and UQCRC2 (*p* < 0.01; Figure 3D,G) and upon WSD for UQCRC2 (Figure 3G) and MTCOI (Figure 3J). ATP5A protein expression decreased over time in EPI WAT (*p* < 0.05; Figure 3N) but was not affected in the ING WAT (Figure 3M). Other complexes of EPI WAT and all complexes in RP WAT remained stable over time and were not affected by the WSD (Figure 3).

### Markers of WAT Browning

**FIGURE 4:**
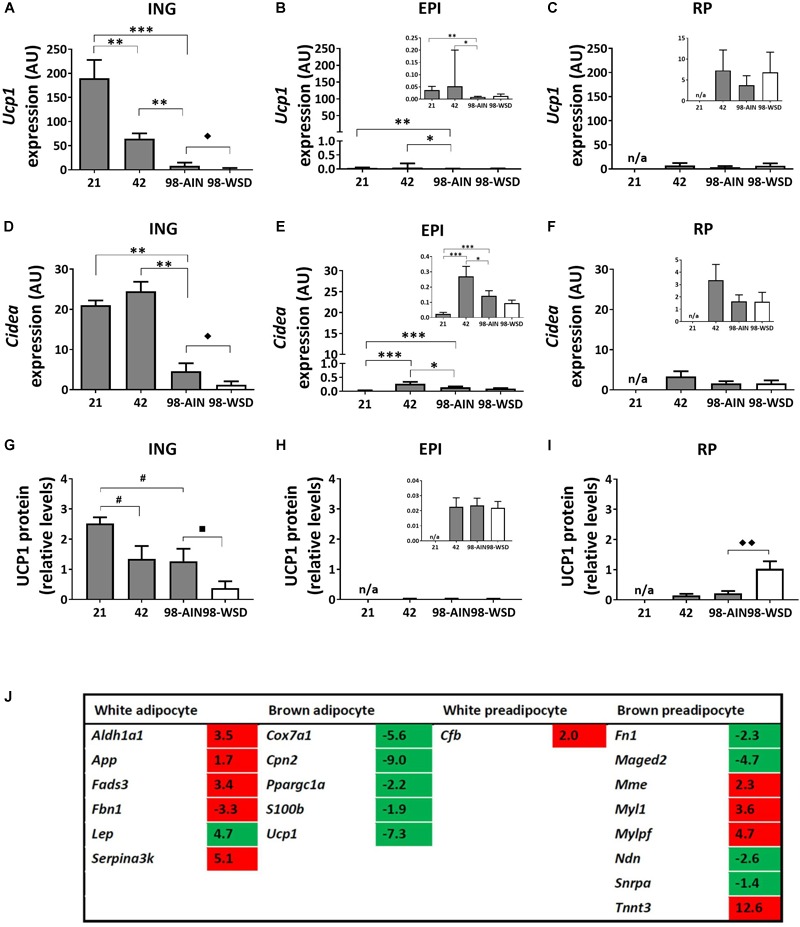
Changes in gene expression of browning markers between PN21 and PN98. *Ucp1* gene expression in **(A)** ING WAT, **(B)** EPI WAT, **(C)** RP WAT; *Cidea* expression in **(D)** ING WAT, **(E)** EPI WAT, **(F)** RP WAT; UCP1 protein levels in **(G)** ING WAT, **(H)** EPI WAT, **(I)** RP WAT; and **(J)** Regulation of genes selective for brown or white (pre)adipocytes over time, analyzed with mRNA sequencing, gene list derived from Gesta et al. (2007) and expression levels of transcripts with *p* < 0.05 included in table. Inserts in figures B, C, E, F, and H show the same data with adapted *y*-axis for better visualization of the low expression data. PN, postnatal day; AIN, AIM93-G diet; WSD, western style diet. Gene expression data expressed as mean + SEM and protein levels as mean levels corrected for total protein (Coomassie staining) + SEM, *n* = 8 for PN 21 and PN42, *n* = 11 for PN98, no data available (n/a) for RP WAT at PN21. Time and WSD effects were analyzed separately; time effect: ^∗^*p* < 0.05; ^∗∗^*p* < 0.01; ^∗∗∗^*p* < 0.001; ^#^0.05 < *p* < 0.1; WSD effect: ^◆^*p* < 0.05; ^◆◆^ *p* < 0.01; ^■^0.05 < *p* < 0.1. Sequence data reported as fold changes between postnatal day 21 over 98. Up regulated values red and down regulated values green.

Gene expression levels of the uncoupling protein *Ucp1*, as marker for browning of WAT, was relatively high in ING WAT at PN21 and decreased substantial over time (*p* < 0.001; Figure 4A). *Ucp1* expression levels were low in visceral depots, but also decreased over time in EPI WAT (*p* < 0.01; Figure 4B,C). *Cidea* gene expression levels were substantially higher in ING WAT compared to visceral depots (Figure 4D,E), but in contrast to *Ucp1* remained stable in ING WAT between PN21 and 42, after which *Cidea* levels declined from PN42 to 98 (*p* < 0.01). UCP1 protein levels tended to decline from PN21 to 42 and 98 (*p* = 0.09; Figure 4G) and were low and not changing over time in the visceral depots (Figure 4H,I). Upon WSD exposure, *Ucp1* and *Cidea* gene expression levels decreased in ING WAT (*p* < 0.05), but remained unaffected in the visceral EPI and RP WAT depots. UCP1 protein levels tended to decline upon the WSD exposure in ING WAT (*p* = 0.1), was not affected by the WSD in the EPI WAT but increased upon the WSD in RP WAT (*p* < 0.01). *Ucp1* and *Cidea* expression levels did not change over time in RP WAT (Figure 4C,F). It should be noted that UCP1 protein levels were very heterogeneous at PN98, showing some animals with much higher levels than on average.

### Depot Differences

ING WAT had overall lower expression of adiposity markers (*Lep, Mest*; *p* < 0.001) and higher levels of mitochondrial (CS and HADH activity and OXPHOS subunits I – IV; *p* < 0.001) and browning markers (*Ucp1* and *Cidea* gene expression and UCP1 protein levels; *p* < 0.001) compared to both EPI and RP WAT. Especially at PN21 and 42 CS and HADH levels were higher in ING compared to EPI and RP WAT (*p* < 0.01) and at PN42 levels of OXPHOS complexes I–IV were also higher in ING compared to RP and EPI WAT (*p* < 0.01). Subsequent decline in CS, HADH and OXPHOS complexes I–IV levels was steeper in ING WAT compared to the visceral depots and resulted in similar CS, HADH and OXPHOS subunit II levels in ING WAT and RP WAT and HADH and subunit II levels being similar in ING WAT and EPI WAT at PN98. *Ucp1* and *Cidea* gene expression levels were also significant higher in ING compared to EPI and RP WAT at PN21 and 42 (*p* < 0.01) and had a steeper decline over time resulting in comparable *Ucp1* gene expression levels in ING and RP WAT at PN98. OXPHOS complex I-IV from the electron transport system, which drives ATP synthesis by OXPHOS complex V, the final step that is bypassed by UCP1 mediated uncoupling. Remarkably, unlike complex I–IV, levels of complex V (represented by ATP5A) were similar between depots.

### Pathway Analysis of mRNA Sequence Data

To better understand the changes in mitochondrial density and function markers in ING WAT over time, mRNA of ING WAT at PN21 and 98 was sequenced (*n* = 4 per time point). 91969 transcripts were found, 23193 of these transcripts had a FPKM value >0 for all samples and an average FPKM > 2 at PN21 or 98. For pathway analysis 5040 transcripts with a *p*-value <0.05 were used, resulting in a list of pathways which were significant regulated over time (Table 3). Most differentially regulated pathways include those involved in regulation of cell proliferation and growth and pathways involved in hormonal regulation and immune response. Protein synthesis was down regulated over time and pathways involved in the FA metabolism were up regulated over time. Overlap between the regulated pathways was plotted as a network map, showing much overlap between pathways, including those involved in the regulation of cell proliferation and growth as well as pathways involved in hormonal regulation and immune response. Two pathways were less connected to the central network: “mitochondrial dysfunction” and “lipid antigen presentation by CD1” (Supplementary Figure 2). The “mitochondrial dysfunction” pathway contained genes representing subunits of the oxidative phosphorylation and other genes involved in the function of mitochondria, and its IPA name follows the general idea that downregulation of the genes contained in this pathway is associated with dysfunctional mitochondria, like in T2D, Alzheimer’s or Parkinson’s disease. Although ingenuity pathway analysis did not indicate a direction for the change in the mitochondrial dysfunction pathway, 35 of the 44 regulated genes in this pathway were down regulated over time, what may indicate that mitochondrial function is reduced at PN98 compared to PN21 in ING WAT (Supplementary Table 1).

**Table 3:** List of significant regulated pathways at PN98 as analyzed by ingenuity pathway analysis (IPA).

Canonical Pathways	-log(*p*-value)	Ratio	*z*-score
**Top 10 down regulated pathways**		
EIF2 signaling	12.10	0.34	-3.833
Estrogen-mediated S-phase entry	5.96	0.58	-3.051
STAT3 pathway	5.58	0.37	-0.577
Cyclins and cell cycle regulation	4.96	0.35	-2.524
Regulation of eIF4 and p70S6K signaling	4.13	0.27	-1.155
mTOR signaling	4.02	0.25	-0.408
Acute phase response signaling	4.00	0.26	-1.333
Phospholipase C signaling	3.78	0.24	-2.359
NF-κB activation by viruses	3.68	0.30	-2.353
14-3-3-mediated signaling	3.61	0.27	-0.378
**Top 10 up regulated pathways**		
Cell cycle: G1/S checkpoint regulation	5.72	0.39	1.706
Aryl hydrocarbon receptor signaling	5.45	0.30	2.000
Protein kinase A signaling	4.71	0.23	1.732
Cell Cycle: G2/M DNA damage checkpoint regulation	4.44	0.39	1.414
Androgen signaling	4.34	0.30	0.535
Sumoylation pathway	4.02	0.30	1.043
PPARα/RXRα activation	3.75	0.25	2.874
LXR/RXR activation	3.20	0.26	0.557
PPAR signaling	2.74	0.27	2.858
Apoptosis signaling	2.67	0.27	0.816
**Top 10 pathways without available activity pattern^∗^**		
Cell cycle control of chromosomal replication	8.08	0.55	NaN
RAR activation	6.75	0.30	NaN
Estrogen receptor signaling	6.15	0.32	NaN
Adipogenesis pathway	6.00	0.31	NaN
Glucocorticoid receptor signaling	5.47	0.25	NaN
Mitochondrial dysfunction	3.87	0.26	NaN
Lipid antigen presentation by CD1	3.85	0.46	NaN
T cell receptor signaling	3.72	0.28	NaN
TR/RXR activation	3.46	0.29	NaN
Assembly of RNA polymerase II complex	3.24	0.34	NaN

*Expression of transcripts determined with mRNA sequencing and data analyzed with ingenuity Pathway Analysis (Qiagen Bioinformatics, Aarhus, Denmark). Difference between postnatal day 21 over 98.^∗^No information on activity pattern was available in IPA and no *Z*-score calculated as a consequence.*

### Targeted Analysis of mRNA Sequence Data

A list of brown and white preadipocyte and adipocyte markers was extracted from literature (Gesta et al., 2007) and checked for their expression in the mRNA sequence data (the 5040 transcripts with *p* < 0.05) to get insight in possible changes in cell populations in ING WAT between PN21 and 98. Expression levels of brown adipocytes markers declined and levels of white adipocytes markers increased from PN21 to 98 (Figure 4J and Supplementary Table 2 for the complete list of markers). For preadipocytes the picture is less clear, some brown preadipocytes markers were up and others were down regulated over time. Of note, the list of markers was extracted from the review of Gesta et al. (2007) and based on results of different experiments, the brown preadipocytes markers which were up regulated over time originate from another experiment than the down regulated markers (Boeuf et al., 2001; Timmons et al., 2007). The list of white preadipocytes markers is short and only one of these genes was abundant in our data set and up regulated over time.

**FIGURE 5:**
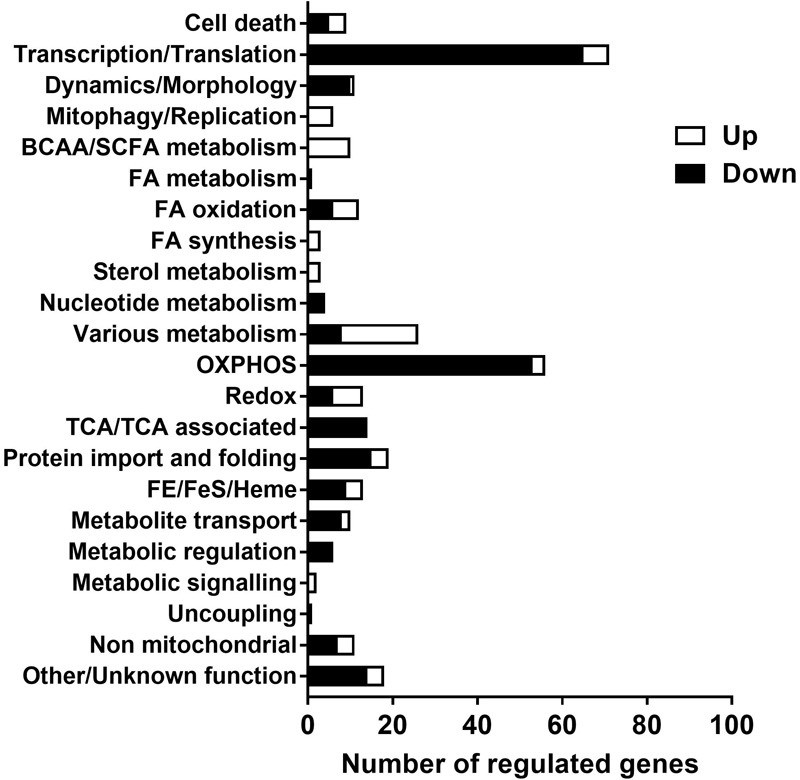
Functional categorization of regulated genes (PN21 over 98) with a strong support for mitochondrial localization. Expression of transcripts analyzed with mRNA sequence, of the transcripts with *p* < 0.05 genes encoding proteins with strong support of mitochondrial localization (Mouse MitoCarta 2.0, Broad institute) were selected and functional categorized with Nextprot (SIB Swiss institute for bioinformatics). The full list of genes with functional categorization is shown in Supplementary Tables 3, 4.

A list of genes of which mitochondrial localization is strongly supported (Mitocarta, 1158 genes) were checked for expression in the data set to further explore functional changes of the mitochondria over time. 327 genes of this list were identified in the data set, of which 231 gene were down regulated, 88 up regulated and 8 genes had discrepancy in regulation between different transcripts. Genes involved in energy metabolism and protein syntheses were down regulated over time, including oxidative phosphorylation, citric acid (TCA) cycle, β-oxidation, import, transport and translation. The up regulated genes showed more diversity in function, including glycolytic metabolism, lipid synthesis, biosynthesis and mitophagy. The list with genes categorized to function is shown in Supplementary Tables 3, 4. A summary of these findings is presented in Figure 5.

Established mitophagy/autophagy markers (Ding and Yin, 2012) were extracted from the mRNA sequence data and their regulation was studied for insight in potential underlying mechanisms of WAT whitening and results are reported in Table 4. Microtubule-associated protein one light chain three alpha (*Map1lc3a*, better known as *Lc3*), sequestosome 1 (*Sqstm1*, better known as *p62*), unk-51 like kinase 2 (*Ulk2*), PTEN induced putative kinase 1 (*Pink1*) and BCL2/adenovirus E1B interacting protein 3 (*Bnip3*) were upregulated at PN98 compared to PN21. To further support this data, LC3 protein levels were analyzed with western blot. LC3.2 protein levels increased between PN21 and 98 in ING and RP WAT and were unchanged in EPI WAT (Supplementary Materials and Supplementary Figure 5). Regrettably, LC3.1 protein levels were not detectable in these samples under these conditions and ratios between LC3.2 and LC3.1 could therefore not be calculated.

**Table 4:** Regulation of mitophagy/autophagy markers in ING WAT from PN21 to 98 as measured with RNA sequencing.

Gene name	ENSBL ID	FC	*p*-value	Adjusted *p*-value
*Map1lc3a (Lc3)*	ENSMUSG00000027602	**1.90**	**0.020**	0.169
*Sqstm1 (p62)*	ENSMUSG00000015837	**1.80**	**0.004**	0.099
*Ulk1*	ENSMUSG00000029512	1.22	0.253	0.466
*Ulk2*	ENSMUSG00000004798	**1.39**	**0.028**	0.190
*Atg7*	ENSMUSG00000030314	1.24	0.100	0.300
*Pink1*	ENSMUSG00000028756	**2.66**	**<0.001**	**0.024**
*Prkn (Parkin)*	ENSMUSG00000023826	Not detected		
*Bnip3l*	ENSMUSG00000022051	Mean count at PN21 < 2		
*Bnip3*	ENSMUSG00000078566	**2.70**	**<0.001**	**0.026**
*Fundc1*	ENSMUSG00000025040	Mean count at PN21 < 2		

*Expression reported as fold changes between postnatal day 21 over 98. Significant differences are bold.*

### Correlations

There were many correlations between mitochondrial density and function markers and WAT weight (Table 5). Specifically, the inverse correlation between CS and HADH levels and weight of the corresponding depot was consistently strong in all depots. ING WAT weight was also inversely correlated to levels of the electron transport system complexes (OXPHOS complexes I–IV). In contrast, no correlation was found between ING WAT weight and the level of OXPHOS complex V (ATP5A; Table 5). RP WAT demonstrated opposite findings: the electron transport system complexes did not correlate with WAT weight, but complex V (ATP5A) showed a mild, inverse correlation with WAT weight. *Lep* gene expression was correlated to WAT weight for each of the depots, but this correlation was stronger in the visceral depots compared to ING WAT. *Ucp1* expression, a key marker for browning of WAT, in ING WAT was correlated to mitochondrial density (CS and mtDNA, Table 5) and function markers (OXPHOS complexes I–IV), but not in RP WAT. In EPI WAT *Ucp1* expression was only to some extent correlated to CS and HADH levels. *Ucp1* expression in ING WAT was not correlated to complex V levels.

**Table 5:** Correlations between WAT weight v. *Ucp1* gene expression and mitochondrial function markers, *Lep* gene expression.

	ING WAT		EPI WAT		RP WAT	
	*r*	*p*	*r*	*p*	*r*	*p*
**Correlations with WAT weight**		
CS activity	-0.738	<0.001	-0.579	<0.001	-0.788	<0.001
HADH activity	-0.759	<0.001	-0.615	<0.001	-0.725	<0.001
mtDNA	-0.444	<0.05	-0.335	0.076	-0.607	<0.01
NDUFB8	-0.681	<0.001	-0.484	<0.01	-0.02	n.s.
SDHB	-0.695	<0.001	-0.451	<0.05	-0.01	n.s.
UQCRC2	-0.734	<0.001	-0.687	<0.001	-0.04	n.s.
MTCOI	-0.585	<0.001	-0.150	n.s.	-0.098	n.s.
ATP5A	-0.091	n.s.	-0.571	<0.001	-0.478	<0.05
*Lep*	0.558	<0.001	0.768	<0.001	0.758	<0.001
**Correlations with *Ucp1***		
CS activity	0.664	<0.001	0.393	<0.05	0.049	n.s.
HADH activity	0.696	<0.001	0.490	<0.01	-0.048	n.s.
mtDNA	0.473	<0.01	0.049	n.s.	-0.136	n.s.
NDUFB8	0.583	<0.001	0.155	n.s.	0.197	n.s.
SDHB	0.582	<0.001	0.113	n.s.	-0.306	n.s.
UQCRC2	0.599	<0.001	0.287	n.s.	-0.182	n.s.
MTCOI	0.511	<0.001	0.031	n.s.	-0.154	n.s.
ATP5A	0.126	n.s.	0.221	n.s.	-0.221	n.s.
*Lep*	-0.402	<0.05	-0.118	n.s.	-0.023	n.s.

## Discussion

In this study, we show clear changes in markers for mitochondrial density, mitochondrial function and browning in ING, EPI, and RP WAT depots of C57BL/6j mice after weaning up to young adulthood. We show that an increase in WAT depot mass and elevated levels of adiposity markers is associated with a decline in mitochondrial density over time in all depots. This decline was depot specific, being most pronounced in ING WAT with the steepest drop from PN21 to 42 and was accelerated by the WSD challenge. RNA sequence data from the ING depot showed that the decline in mitochondrial density was accompanied by a transfer from active, ATP producing mitochondria with a clear uncoupling potential toward mitochondria with a more diverse metabolic function and a higher biosynthesis. The RNA sequence data also showed that ING WAT developed from a “browner” toward a “whiter” phenotype with increased coupled mitochondria. These data also show that this is accompanied by an increased mRNA expression of mitophagy markers.

The present study aimed to comprehensively compare postweaning changes of markers for mitochondrial function and WAT browning in three different WAT depots. There is evidence from one other study showing decreasing mitochondrial enzyme activity in human subcutaneous WAT from the postnatal period to adulthood (Novak et al., 1973), which is in accordance with the findings in our study. Previous experiments in rats showed lower mitochondrial respiration, enzyme activity and mitochondrial abundance in ING compared to EPI WAT, both when measured in whole tissue or isolated adipocytes (Prunet-Marcassus et al., 1999; Deveaud et al., 2004). But a study in adult mice showed a higher mitochondrial performance in isolated mitochondria of ING compared to EPI WAT while mitochondrial density was not different between the depots (Schottl et al., 2015). In the present study respiration was not measured, but the results were more in agreement with the later study, showing higher mitochondrial enzyme, OXPHOS protein and RNA marker levels in ING compared to EPI WAT of young mice. Moreover, the results of this study showed that the differences in mitochondrial density and function markers between ING and visceral WAT are age specific, being prominent at weaning and much smaller in early adulthood.

The declining mitochondrial density and function markers from weaning to young adulthood in ING WAT was accompanied by a diminished gene expression of the WAT browning markers *Ucp1* and *Cidea.* UCP1 proteins levels tended to decrease accordingly, although statistical significance was not reached for the older AIN93 animals. Subcutaneous depots are more prone to WAT browning than visceral depots and have a higher abundancy of adipocytes with a brown-like phenotype, that can be activated upon cold exposure (Wu et al., 2012). The higher levels of WAT browning markers at weaning may therefore be part of a protective response of the vulnerable pups to the colder environment in the postnatal period, comparable to the uncoupling response in the brown adipose tissue (Obregon et al., 1989). Indeed, the decreased expression of brown adipocyte markers in the RNA sequence data at PN98 suggests that abundance of brown or brown-like adipocytes, with the capacity to produce heat, is declining over time. This is in line with evidence of other mice studies showing browning of the WAT depots from PN10 to 20 and subsequent whitening from PN20 to 30 (Xue et al., 2007; Lasar et al., 2013; Birnbacher et al., 2018) a process that has shown to be strongly genetically controlled (Chabowska-Kita et al., 2015). The increased abundancy of white adipocytes, with their lipid storage and insulation capacity, at the same time point further supports this explanation. This also provides an explanation for the differences between subcutaneous and visceral WAT, of which the latter showed a much lower expression of the browning makers *Ucp1* and *Cidea*. Again, this it is fully in line with the developmental needs of a young pup, being small and vulnerable to cold stress, to a large, more mature individual with a sufficient layer of thermal insulation provided by WAT. This notion is supported by the substantial decline in preadipocyte marker expression from PN21 to PN42 and the increased expression of white adipocyte markers at PN98. The latter indicates that preadipocytes differentiated to adipocytes between those time points, a process that is probably already ongoing at PN21. Indeed, the decline in preadipocyte marker expression is previously reported and there coincided with an increased expression of adipocyte marker expression (Xue et al., 2007). Environmental factors, like dietary interventions or early life stress (Yam et al., 2017), may change the pace of whitening and subsequently have long-lasting effects on the oxidative and storage capacity of WAT depots. It would be of interest to investigate the effects of temperature on postweaning changes, in particular by repeating the experiment under thermoneutrality, but also at intermediate and lower low-ambient temperatures, and what the consequence of changes in the pace of whitening of the WAT depots is for later life metabolic health and WAT function. Moreover, investigating the pace of whitening and subsequent later life health consequences in UCP1 knockout or other relevant genetic mouse models or investigating the consequences of different aspects of the weaning process (maternal separation, dietary switch and early/late weaning) can give insight in the underlying mechanisms.

Recent publications revealed that the whitening of WAT is controlled by autophagy induced mitochondrial clearance (mitophagy), indicated by the activation of mitophagy during the beige to white transition in cultured adipocytes following β3-AR agonist withdrawal and the impaired whitening when autophagy is deleted in knock-out mouse models (Altshuler-Keylin et al., 2016; Lu et al., 2019). Therefore, we checked the regulation of genes known to be involved in mitophagy (Ding and Yin, 2012) in ING WAT between PN21 and 98, the depot where changes in mitochondrial abundance and expression of browning markers was biggest. Indeed, genes involved in mitophagy were upregulated over time, as was the protein level of the autophagy marker LC3.2, confirming the role of mitophagy in whitening of WAT depots.

Our data on mitochondrial metabolic pathways show that mitochondria develop from organelles with a high expression of pathways directed at energy production (and dissipation), as in brown adipocytes (Forner et al., 2009), toward coupled mitochondria which display a wider variety of biochemical pathways. Part of the changes may be related to maturation of ING WAT, since expression of proteins involved in fatty acid metabolism and of mitochondrial chaperones has been shown to increase during adipogenesis (Wilson-Fritch et al., 2003). In ING WAT of the more mature PN98 mice we observe a much smaller number of genes related to especially protein import, translation and OXPHOS as well as nucleotide metabolism, suggestion a decrease in mitochondrial “growth”/biogenesis, while genes related to lipid synthesis, branched chain amino acid/short chain fatty acid metabolism, steroid metabolism and redox signaling appear, as well as a substantial number of genes related to diverse biosynthetic pathways (Figure 5). This indicates that the mitochondria have reached a condition where they interact more with the rest of the cell, no longer unilaterally focused on growth and energy metabolism (and dissipation) only. This especially suggests that ING WAT mitochondria have reached a steady state, which is supported by the appearance of autophagy and apoptosis genes, essential for mitochondrial (and cellular) turnover and quality control (Zimmermann and Reichert, 2017). Our data further indicate that ING WAT between PN21 (and possibly earlier) and PN42 provides a physiological relevant model to study and better understand functional changes in mitochondria related to adipose tissue development and mitochondrial adaptive capacity and to understand mitochondrial changes related to WAT whitening.

The changes in the functionality of the mitochondria in the WAT depots and the remodeling of these depots in early life may have an impact for intervention studies starting in early life, as the effect of the intervention may be very dependent on the starting point of the intervention. In line with the developmental origins of health and disease theory (Hales and Barker, 2001; Gluckman and Hanson, 2004), developmental processes may respond in a manner to optimally match an individual to anticipate later life conditions (Hanson and Gluckman, 2014). Previous studies in our lab showed that the postnatal period is amendable to nutritional programming since a relative mild dietary intervention at weaning indeed increased levels of later life mitochondrial oxidative capacity (Kodde et al., 2017). Although the perspective is there, additional studies are needed to fully understand to which extent nutritional interventions in the timeframe of weaning provides a window of opportunity for protection against later life metabolic disease.

## Conclusion

The present study showed a decline in mitochondrial density and oxidative capacity markers in WAT depots of young C57Bl/6j mice over time while adipose tissue mass increased in size. The decline is more explicit in ING WAT compared to the visceral depots and is accompanied by an evolution from a browner, energy dissipating, to a whiter, biosynthetic, adipose tissue phenotype. These developmental changes may provide an opportunity to program a healthy WAT mitochondrial phenotype by nutritional interventions during the weaning period.

## Ethics Statement

All animal procedures were in accordance with the principles of good laboratory animal care following the EU directive for the protection of animals used for scientific purposes and approved by an external, independent Animal Experimental Committee (DEC consult, Soest, Netherlands).

## Author Contributions

AK, EE, and KvL conducted the experiments. AK and JK analyzed the data and drafted the manuscript. AK, AO, and JK interpreted the results. AK prepared the figures. All authors contributed to the design of the study, edited and revised the manuscript, and approved the final version of the manuscript.

## Conflict of Interest Statement

AK, EE, AO, KvL, and EvdB are employees of the Danone Nutricia Research. The remaining author declares that the research was conducted in the absence of any commercial or financial relationships that could be construed as a potential conflict of interest.
