# Supplementary material for: Maturation of White Adipose Tissue Function in C57BL/6j Mice From Weaning to Young Adulthood
Source: Front Physiol. 2019 Jul 9;10:836. doi: 10.3389/fphys.2019.00836 (PMC6629938; doi:10.3389/fphys.2019.00836)
Supplement: Supplementary file 1 [file Table_1.docx]

***Supplementary Material***

**Maturation of White Adipose Tissue Function in C57BL/6j Mice from Weaning to Young Adulthood**

**Andrea Kodde^*^, Eefje Engels, Annemarie Oosting, Kees van Limpt, Eline M. van der Beek, Jaap Keijer**

*** Correspondence:** Andrea Kodde: andrea.kodde@danone.com

**Supplementary methods: LC3 protein levels**

Protein levels of LC3 as marker for mitophagy, were measured with western blot (1). Briefly, 15 μg of total protein per sample was used for SDS-PAGE, transfer to a PVDF membrane was done using the Pierce Power Blot (Thermo Fisher Scientific). Blots were blocked with 5% Protifar (Nutricia, Zoetermeer, The Netherlands), incubated with goat anti mouse LC3 (Nanotools, Teningen, Germany) as primary antibody and HRP conjugated anti mouse IgG (R&D systems) as a secondary antibody. LC3 protein levels were detected with Supersignal West Femto (Thermo Fisher Scientific) and were related to total protein levels as analyzed by Coomassie brilliant blue staining. Protein levels were detected with the Chemidoc XRS and analyzed by Quantity One (Biorad). Results and a representative Western blot stained for LC3 and the corresponding Coomassie brilliant blue stained blot is shown in Supplementary figure 5.

**Supplementary Table 1:** Expression of transcripts belonging to mitochondrial dysfunction pathway as determined with mRNA sequencing and data analyzed with ingenuity Pathway Analysis (Qiagen Bioinformatics, Aarhus, Denmark). Difference between postnatal day 21 over 98.

| Gene ID | Ensembl ID | p-value | FDR | FC |
| --- | --- | --- | --- | --- |
| *Acox2* | ENSMUST00000126352 | 0,047 | 0,226 | -2,00 |
| *Aph1a* | ENSMUST00000015894 | 0,009 | 0,131 | -1,50 |
| *Aph1b* | ENSMUST00000034934 | 0,011 | 0,140 | 1,66 |
| *App* | ENSMUST00000005406 | 0,049 | 0,228 | 1,74 |
| *Atp5a1* | ENSMUST00000114748 | 0,004 | 0,100 | -2,00 |
| *Atp5b* | ENSMUST00000125992 | 0,029 | 0,192 | -4,30 |
| *Atp5d* | ENSMUST00000105366 | 0,019 | 0,167 | -1,90 |
| *Atp5e* | ENSMUST00000149191 | 0,001 | 0,048 | -2,20 |
| *Atp5g1* | ENSMUST00000107684 | 0,035 | 0,205 | -1,70 |
| *Atp5g3* | ENSMUST00000131045 | 0,048 | 0,227 | -1,50 |
| *Atp5h* | ENSMUST00000138779 | 0,026 | 0,185 | -1,70 |
| *Atp5o* | ENSMUST00000023677 | 0,006 | 0,117 | -2,60 |
| *Bace2* | ENSMUST00000047275 | 0,021 | 0,172 | -3,00 |
| *Cat* | ENSMUST00000028610 | 0,001 | 0,062 | 2,24 |
| *Cox10* | ENSMUST00000049091 | 0,002 | 0,074 | -2,10 |
| *Cox11* | ENSMUST00000020851 | 0,003 | 0,083 | -2,10 |
| *Cox15* | ENSMUST00000045562 | 0,021 | 0,173 | -1,60 |
| *Cox4i2* | ENSMUST00000010020 | 0,043 | 0,220 | -2,00 |
| *Cox5a* | ENSMUST00000000090 | 0,040 | 0,215 | -2,00 |
| *Cox6b2* | ENSMUST00000182272 | 0,020 | 0,168 | -2,60 |
| *Cox7a1* | ENSMUST00000098594 | 0,015 | 0,154 | -5,60 |
| *Cyb5a* | ENSMUST00000025549 | 0,002 | 0,072 | 2,19 |
| *Cyc1* | ENSMUST00000023210 | 0,042 | 0,219 | -1,80 |
| *Glrx2* | ENSMUST00000152435 | 0,016 | 0,159 | -1,80 |
| *Gpx7* | ENSMUST00000030332 | 0,049 | 0,228 | -1,80 |
| *Htra2* | ENSMUST00000113962 | 0,032 | 0,198 | -1,50 |
| *Maoa* | ENSMUST00000026013 | 0,002 | 0,075 | 1,97 |
| *Maob* | ENSMUST00000040820 | 0,023 | 0,177 | 1,67 |
| *Ndufa3* | ENSMUST00000108644 | 0,049 | 0,229 | -1,80 |
| *Ndufa11* | ENSMUST00000002452 | 0,035 | 0,205 | -1,80 |
| *Ndufb5* | ENSMUST00000122290 | 0,045 | 0,222 | -1,80 |
| *Ndufb10* | ENSMUST00000141324 | 0,026 | 0,186 | -2,30 |
| *Ndufs7* | ENSMUST00000155523 | 0,033 | 0,200 | -1,60 |
| *Ndufs8* | ENSMUST00000075092 | 0,017 | 0,161 | -1,90 |
| *Pdha1* | ENSMUST00000156531 | 0,029 | 0,192 | -3,30 |
| *Pink1* | ENSMUST00000030536 | 0,000 | 0,024 | 2,66 |
| *Rhot2* | ENSMUST00000043897 | 0,045 | 0,223 | -1,50 |
| *Sdhb* | ENSMUST00000010007 | 0,045 | 0,223 | -1,90 |
| *Sdhd* | ENSMUST00000000175 | 0,047 | 0,226 | -1,80 |
| *Surf1* | ENSMUST00000150776 | 0,007 | 0,121 | 3,54 |
| *Txn2* | ENSMUST00000174468 | 0,014 | 0,152 | -4,00 |
| *Uqcr11* | ENSMUST00000141683 | 0,048 | 0,227 | -3,50 |
| *Uqcrfs1* | ENSMUST00000042834 | 0,046 | 0,224 | -1,90 |
| *Xdh* | ENSMUST00000024866 | 0,004 | 0,101 | 1,84 |

FC: Fold change; FDR: Fold discovery rate

**Supplementary Table 2:** Regulation of genes selective for brown or white (pre)adipocytes over time, analysed with mRNA sequencing. Gene list derived from (2) and expression levels of transcripts with p<0.05 included in table. Expression reported as fold changes between postnatal day 21 over 98. Up regulated values red and down regulated values green, empty cells indicate that these genes were not significantly regulated.

| **Preadipocyte markers:** | | | **Adipocyte markers** | | |
| --- | --- | --- | --- | --- | --- |
| **Gene** | **Time** |  | **Gene** | **Time** |  |
| **Brown adipose tissue** | | | **Brown adipose tissue** | | |
| *Acta1* |  |  | *Acaa2* |  |  |
| *Actc1* |  |  | *Acss1* |  |  |
| *Actn4* |  |  | *Cidea* |  |  |
| *Adam15* |  |  | *Cox7a1* | -5.6 |  |
| *Cd83* |  |  | *Cox8b* |  |  |
| *Chrna1* |  |  | *Cpn2* | -9.0 |  |
| *Cldn5* |  |  | *Cpt1b* |  |  |
| *Fn1* | -2.3 |  | *Dio2* |  |  |
| *Hdlbp* |  |  | *Elovl3* |  |  |
| *Icam2* |  |  | *Elovl6* |  |  |
| *Maged2* | -4.7 |  | *Esrrg* |  |  |
| *Mme* | 2.3 |  | *Fgf16* |  |  |
| *Myh3* |  |  | *Gpd2* |  |  |
| *Myl1* | 3.6 |  | *Hoxa1* |  |  |
| *Mylpf* | 4.7 |  | *Hoxc4* |  |  |
| *Myog* |  |  | *Mpzl2* |  |  |
| *Ndn* | -2.6 |  | *Ntrk3* |  |  |
| *Snrpa* | -1.4 |  | *Otop1* |  |  |
| *Tnnc1* |  |  | *Ppara* |  |  |
| *Tnni1* |  |  | *Ppargc1a* | -2.2 |  |
| *Tnnt3* | 12.6 |  | *S100b* | -1.9 |  |
|  |  |  | *Sirt3* |  |  |
|  |  |  | *Ucp1* | -7.3 |  |
| **White adipose tissue** | | | **White adipose tissue** | | |
| *C2* |  |  | *Aldh1a1* | 3.5 |  |
| *C3* |  |  | *App* | 1.7 |  |
| *Cfb* | 2.0 |  | *Ccl6* |  |  |
| *Fads2* |  |  | *Dpt* |  |  |
|  |  |  | *Fads3* | 3.4 |  |
|  |  |  | *Fbn1* | -3.3 |  |
|  |  |  | *Hmgn3* |  |  |
|  |  |  | *Hoxa4* |  |  |
|  |  |  | *Hoxa7* |  |  |
|  |  |  | *Hoxc8* |  |  |
|  |  |  | *Lep* | 4.7 |  |
|  |  |  | *Nrip1* |  |  |
|  |  |  | *Psat1* |  |  |
|  |  |  | *Retn* |  |  |
|  |  |  | *Retnla* |  |  |
|  |  |  | *Serpina3k* | 5.1 |  |
|  |  |  | *Sphk1* |  |  |

**Supplementary Table 3:** Functional categorization of genes downregulated at postnatal day 98 compared to 21, with strong support of mitochondrial localization. Expression of transcripts analyzed with mRNA sequence, of the transcripts with p<0.05 genes encoding proteins with strong support of mitochondrial localization (Mouse MitoCarta 2.0, Broad institute) were selected and functional categorized with Nextprot (SIB Swiss institute for bioinformatics)

| **Functional Category** | **Symbol** | **EnsemblGeneID** | **FC** | **P.Value** |
| --- | --- | --- | --- | --- |
| Cell death | Prelid1 | ENSMUSG00000021486 | -7,5 | 0,002 |
| Cell death | **Endog** | ENSMUSG00000015337 | -2 | 0,030 |
| Cell death | Fkbp8 | ENSMUSG00000019428 | -1,8 | 0,032 |
| Cell death | Alkbh7 | ENSMUSG00000002661 | -1,8 | 0,036 |
| Cell death | Aifm2 | ENSMUSG00000020085 | -1,4 | 0,017 |
| Dynamics/Morphology | **Stoml2** | ENSMUSG00000028455 | -1,4 | 0,038 |
| Dynamics/Morphology | **Opa1** | ENSMUSG00000038084 | -2,4 | 0,023 |
| Dynamics/Morphology | Mul1 | ENSMUSG00000041241 | -1,9 | 0,019 |
| Dynamics/Morphology | **Rab24** | ENSMUSG00000034789 | -1,8 | 0,018 |
| Dynamics/Morphology | **Atad3a** | ENSMUSG00000029036 | -1,8 | 0,017 |
| Dynamics/Morphology | Cisd2 | ENSMUSG00000028165 | -1,7 | 0,016 |
| Dynamics/Morphology | **Letm1** | ENSMUSG00000005299 | -1,6 | 0,040 |
| Dynamics/Morphology | **Poldip2** | ENSMUSG00000001100 | -1,6 | 0,015 |
| Dynamics/Morphology | **Opa3** | ENSMUSG00000052214 | -1,5 | 0,026 |
| Dynamics/Morphology | **Mcu** | ENSMUSG00000009647 | -1,4 | 0,022 |
| FA oxidation | **Etfb** | ENSMUSG00000004610 | -2,3 | 0,027 |
| FA oxidation | **Etfdh** | ENSMUSG00000027809 | -2,2 | 0,002 |
| FA oxidation | **Acadl** | ENSMUSG00000026003 | -2,1 | 0,036 |
| FA oxidation | Slc25a29 | ENSMUSG00000021265 | -1,7 | 0,024 |
| FA oxidation | Tysnd1 | ENSMUSG00000020087 | -1,6 | 0,015 |
| FA oxidation | **Lactb** | ENSMUSG00000032370 | -1,4 | 0,022 |
| Fe/FeS/Heme | **Nfu1** | ENSMUSG00000029993 | -120,5 | 0,037 |
| Fe/FeS/Heme | **Cisd3** | ENSMUSG00000078695 | -2,2 | 0,018 |
| Fe/FeS/Heme | **Alas1** | ENSMUSG00000032786 | -1,9 | 0,021 |
| Fe/FeS/Heme | **Fxn** | ENSMUSG00000059363 | -1,8 | 0,026 |
| Fe/FeS/Heme | **Isca1** | ENSMUSG00000044792 | -1,7 | 0,025 |
| Fe/FeS/Heme | **Ppox** | ENSMUSG00000062729 | -1,7 | 0,021 |
| Fe/FeS/Heme | Slc25a37 | ENSMUSG00000034248 | -1,7 | 0,036 |
| Fe/FES/Heme | Rsad1 | ENSMUSG00000039096 | -1,6 | 0,017 |
| Fe/FeS/Heme | **Iba57** | ENSMUSG00000049287 | -1,5 | 0,044 |
| Metabolic regulation | Sirt4 | ENSMUSG00000029524 | -2,8 | 0,003 |
| Metabolic regulation | **Agk** | ENSMUSG00000029916 | -1,7 | 0,012 |
| Metabolic regulation | **Agpat5** | ENSMUSG00000031467 | -1,6 | 0,017 |
| Metabolic regulation | **Gadd45gip1** | ENSMUSG00000033751 | -1,6 | 0,047 |
| Metabolic regulation | Rdh11 | ENSMUSG00000066441 | -1,5 | 0,025 |
| Metabolic regulation | Pick1 | ENSMUSG00000068206 | -1,5 | 0,031 |
| Metabolite transport | **Slc25a10** | ENSMUSG00000025792 | -5000 | 0,003 |
| Metabolite transport | **Slc25a42** | ENSMUSG00000002346 | -4,3 | 0,002 |
| Metabolite transport | **Slc25a45** | ENSMUSG00000024818 | -3 | 0,028 |
| Metabolite transport | **Slc25a35** | ENSMUSG00000018740 | -2,6 | 0,008 |
| Metabolite transport | **Slc25a19** | ENSMUSG00000020744 | -2,6 | 0,013 |
| Metabolite transport | **Slc25a3** | ENSMUSG00000061904 | -1,9 | 0,016 |
| Metabolite transport | **Slc25a5** | ENSMUSG00000016319 | -1,7 | 0,011 |
| Metabolite transport | Slc16a1 | ENSMUSG00000032902 | -1,7 | 0,018 |
| Non mitochondrial | Rpl10a | ENSMUSG00000037805 | -3 | 0,002 |
| Non mitochondrial | Tcirg1 | ENSMUSG00000001750 | -3 | 0,032 |
| Non mitochondrial | **Lamc1** | ENSMUSG00000026478 | -2,2 | 0,007 |
| Non mitochondrial | Rps15a | ENSMUSG00000008683 | -2,2 | 0,048 |
| Non mitochondrial | Rpia | ENSMUSG00000053604 | -1,9 | 0,002 |
| Non mitochondrial | Rpl34 | ENSMUSG00000062006 | -1,8 | 0,040 |
| Non mitochondrial | **Rps18** | ENSMUSG00000008668 | -1,4 | 0,026 |
| Nucleotide metabolism | Apex2 | ENSMUSG00000025269 | -2,9 | 0,001 |
| Nucleotide metabolism | **Dut** | ENSMUSG00000027203 | -2,6 | 0,002 |
| Nucleotide metabolism | Nt5c | ENSMUSG00000020736 | -2,6 | 0,012 |
| Nucleotide metabolism | **Nme4** | ENSMUSG00000024177 | -2,1 | 0,026 |
| Other | Nt5dc2 | ENSMUSG00000071547 | -6,2 | 0,000 |
| Other | **Glod4** | ENSMUSG00000017286 | -3,3 | 0,008 |
| Other | **Hdhd3** | ENSMUSG00000038422 | -3 | 0,006 |
| Other | **Tha1** | ENSMUSG00000017713 | -2,6 | 0,000 |
| Other | **Abhd11** | ENSMUSG00000040532 | -2,5 | 0,009 |
| Other | **1700021F05Rik** | ENSMUSG00000019797 | -2,1 | 0,028 |
| Other | **2010107E04Rik** | ENSMUSG00000021290 | -1,8 | 0,028 |
| Other | **2310061I04Rik** | ENSMUSG00000050705 | -1,8 | 0,016 |
| Other | **Abhd10** | ENSMUSG00000033157 | -1,5 | 0,022 |
| Other | Xrcc6bp1 | ENSMUSG00000025436 | -1,4 | 0,025 |
| Other | **Dhrs1** | ENSMUSG00000002332 | -1,4 | 0,026 |
| Other | Rcc1l | ENSMUSG00000061979 | -1,3 | 0,048 |
| OXPHOS | **Sdhc** | ENSMUSG00000058076 | -8,4 | 0,000 |
| OXPHOS | **Cox7a1** | ENSMUSG00000074218 | -5,6 | 0,015 |
| OXPHOS | **Uqcr11** | ENSMUSG00000020163 | -3,5 | 0,048 |
| OXPHOS | **Cmc2** | ENSMUSG00000014633 | -3,5 | 0,001 |
| OXPHOS | Cep89 | ENSMUSG00000023072 | -3,1 | 0,029 |
| OXPHOS | **Uqcc1** | ENSMUSG00000005882 | -2,6 | 0,024 |
| OXPHOS | Cox6b2 | ENSMUSG00000051811 | -2,6 | 0,020 |
| OXPHOS | **Atp5k** | ENSMUSG00000050856 | -2,6 | 0,004 |
| OXPHOS | **Cox7c** | ENSMUSG00000017778 | -2,3 | 0,010 |
| OXPHOS | **Ndufb10** | ENSMUSG00000040048 | -2,2 | 0,010 |
| OXPHOS | **Atp5e** | ENSMUSG00000016252 | -2,2 | 0,001 |
| OXPHOS | **Fmc1** | ENSMUSG00000019689 | -2,2 | 0,018 |
| OXPHOS | Cox10 | ENSMUSG00000042148 | -2,1 | 0,002 |
| OXPHOS | **Adck4** | ENSMUSG00000003762 | -2,1 | 0,050 |
| OXPHOS | Atp5sl | ENSMUSG00000057229 | -2 | 0,001 |
| OXPHOS | **Tomm5** | ENSMUSG00000078713 | -2 | 0,013 |
| OXPHOS | **Coa7** | ENSMUSG00000048351 | -2 | 0,001 |
| OXPHOS | **Cox5a** | ENSMUSG00000000088 | -2 | 0,040 |
| OXPHOS | **Cox4i2** | ENSMUSG00000009876 | -2 | 0,043 |
| OXPHOS | **Atp5b** | ENSMUSG00000025393 | -2 | 0,000 |
| OXPHOS | **Atp5a1** | ENSMUSG00000025428 | -2 | 0,004 |
| OXPHOS | **Tbrg4** | ENSMUSG00000000384 | -2 | 0,013 |
| OXPHOS | Fastk | ENSMUSG00000028959 | -2 | 0,006 |
| OXPHOS | **Hccs** | ENSMUSG00000031352 | -1,9 | 0,007 |
| OXPHOS | **Ndufs1** | ENSMUSG00000025968 | -1,9 | 0,010 |
| OXPHOS | **Ndufs8** | ENSMUSG00000059734 | -1,9 | 0,017 |
| OXPHOS | **Sdhb** | ENSMUSG00000009863 | -1,9 | 0,045 |
| OXPHOS | **Uqcrfs1** | ENSMUSG00000038462 | -1,9 | 0,046 |
| OXPHOS | Coa4 | ENSMUSG00000044881 | -1,9 | 0,020 |
| OXPHOS | **Atp5d** | ENSMUSG00000003072 | -1,9 | 0,019 |
| OXPHOS | **Atpif1** | ENSMUSG00000054428 | -1,9 | 0,025 |
| OXPHOS | **Cyc1** | ENSMUSG00000022551 | -1,8 | 0,042 |
| OXPHOS | **Ndufb5** | ENSMUSG00000027673 | -1,8 | 0,044 |
| OXPHOS | Ndufc1 | ENSMUSG00000037152 | -1,8 | 0,047 |
| OXPHOS | **Ndufa3** | ENSMUSG00000035674 | -1,8 | 0,049 |
| OXPHOS | **Sdhd** | ENSMUSG00000000171 | -1,8 | 0,047 |
| OXPHOS | **Uqcrh** | ENSMUSG00000063882 | -1,8 | 0,037 |
| OXPHOS | **Lace1** | ENSMUSG00000038302 | -1,8 | 0,047 |
| OXPHOS | **Ndufaf4** | ENSMUSG00000028261 | -1,7 | 0,038 |
| OXPHOS | **Ndufc2** | ENSMUSG00000030647 | -1,7 | 0,040 |
| OXPHOS | **Minos1** | ENSMUSG00000050608 | -1,7 | 0,030 |
| OXPHOS | **Coq4** | ENSMUSG00000026798 | -1,7 | 0,042 |
| OXPHOS | Atp5g1 | ENSMUSG00000006057 | -1,7 | 0,035 |
| OXPHOS | Pdss2 | ENSMUSG00000038240 | -1,6 | 0,048 |
| OXPHOS | **Ndufb11** | ENSMUSG00000031059 | -1,6 | 0,004 |
| OXPHOS | **Ndufs7** | ENSMUSG00000020153 | -1,6 | 0,033 |
| OXPHOS | **Atp5c1** | ENSMUSG00000025781 | -1,6 | 0,004 |
| OXPHOS | **Acad9** | ENSMUSG00000027710 | -1,5 | 0,023 |
| OXPHOS | **Coa3** | ENSMUSG00000017188 | -1,5 | 0,032 |
| OXPHOS | **Atp5f1** | ENSMUSG00000000563 | -1,5 | 0,043 |
| OXPHOS | Atp5g3 | ENSMUSG00000018770 | -1,5 | 0,048 |
| OXPHOS | **Ttc19** | ENSMUSG00000042298 | -1,5 | 0,045 |
| OXPHOS | **Noa1** | ENSMUSG00000036285 | -1,5 | 0,004 |
| Protein import and folding | Dnajc4 | ENSMUSG00000024963 | -3 | 0,002 |
| Protein import and folding | **Ppif** | ENSMUSG00000021868 | -2,8 | 0,001 |
| Protein import and folding | **Timm13** | ENSMUSG00000020219 | -2,4 | 0,000 |
| Protein import and folding | **Timm8a1** | ENSMUSG00000045455 | -2,2 | 0,002 |
| Protein import and folding | **Timm17b** | ENSMUSG00000031158 | -1,9 | 0,004 |
| Protein import and folding | **Tomm40** | ENSMUSG00000002984 | -1,9 | 0,009 |
| Protein import and folding | **Mtx1** | ENSMUSG00000064068 | -1,9 | 0,008 |
| Protein import and folding | **Timm50** | ENSMUSG00000003438 | -1,8 | 0,030 |
| Protein import and folding | **Hspd1** | ENSMUSG00000025980 | -1,8 | 0,014 |
| Protein import and folding | **Tmem14c** | ENSMUSG00000021361 | -1,7 | 0,019 |
| Protein import and folding | **Oxa1l** | ENSMUSG00000000959 | -1,7 | 0,004 |
| Protein import and folding | **Tomm22** | ENSMUSG00000022427 | -1,5 | 0,014 |
| Protein import and folding | **Tmem186** | ENSMUSG00000043140 | -1,4 | 0,012 |
| Protein import and folding | Abcb9 | ENSMUSG00000029408 | -1,4 | 0,027 |
| Protein import and folding | Grpel2 | ENSMUSG00000024580 | -1,4 | 0,032 |
| Redox | **Txn2** | ENSMUSG00000005354 | -4 | 0,014 |
| Redox | **Prdx4** | ENSMUSG00000025289 | -2,2 | 0,006 |
| Redox | **Ptges2** | ENSMUSG00000026820 | -1,9 | 0,024 |
| Redox | Glrx2 | ENSMUSG00000018196 | -1,8 | 0,016 |
| Redox | **Selo** | ENSMUSG00000035757 | -1,7 | 0,009 |
| Redox | Txndc12 | ENSMUSG00000028567 | -1,5 | 0,027 |
| TCA/TCA associated | **Pdha1** | ENSMUSG00000031299 | -3,3 | 0,029 |
| TCA/TCA associated | **Gls** | ENSMUSG00000026103 | -3 | 0,003 |
| TCA/TCA associated | Mmadhc | ENSMUSG00000026766 | -2,8 | 0,036 |
| TCA/TCA associated | **Mecr** | ENSMUSG00000028910 | -2,4 | 0,043 |
| TCA/TCA associated | **Suclg1** | ENSMUSG00000052738 | -2,1 | 0,019 |
| TCA/TCA associated | **Aco2** | ENSMUSG00000022477 | -2 | 0,047 |
| TCA/TCA associated | **Sucla2** | ENSMUSG00000022110 | -2 | 0,036 |
| TCA/TCA associated | **Got2** | ENSMUSG00000031672 | -1,9 | 0,002 |
| TCA/TCA associated | **Me2** | ENSMUSG00000024556 | -1,8 | 0,044 |
| TCA/TCA associated | **Pck2** | ENSMUSG00000040618 | -1,6 | 0,037 |
| TCA/TCA associated | **Nit2** | ENSMUSG00000022751 | -1,6 | 0,021 |
| TCA/TCA associated | **Idh3g** | ENSMUSG00000002010 | -1,4 | 0,044 |
| TCA/TCA associated | Gls2 | ENSMUSG00000044005 | -1,4 | 0,050 |
| TCA/TCA associated | **Slc25a11** | ENSMUSG00000014606 | -1,4 | 0,035 |
| Transcription | **Mterfd3** | ENSMUSG00000049038 | -2 | 0,019 |
| Transcription | **Supv3l1** | ENSMUSG00000020079 | -1,9 | 0,030 |
| Transcription | **Polrmt** | ENSMUSG00000020329 | -1,8 | 0,028 |
| Transcription | Ptcd2 | ENSMUSG00000021650 | -1,8 | 0,004 |
| Translation | **Rmnd1** | ENSMUSG00000019763 | -3,7 | 0,001 |
| Translation | Fkbp10 | ENSMUSG00000001555 | -3,7 | 0,002 |
| Translation | Hemk1 | ENSMUSG00000032579 | -3,4 | 0,007 |
| translation | Alkbh1 | ENSMUSG00000079036 | -2,9 | 0,014 |
| Translation | Gtpbp3 | ENSMUSG00000007610 | -2,6 | 0,029 |
| Translation | **Fastkd2** | ENSMUSG00000025962 | -2,5 | 0,004 |
| Translation | **Qrsl1** | ENSMUSG00000019863 | -2,3 | 0,000 |
| Translation | **Mrps28** | ENSMUSG00000040269 | -2,3 | 0,006 |
| Translation | **Dap3** | ENSMUSG00000068921 | -2,2 | 0,015 |
| Translation | **Elac2** | ENSMUSG00000020549 | -2,2 | 0,007 |
| Translation | **Mrps12** | ENSMUSG00000045948 | -2,1 | 0,000 |
| Translation | **Mrps36** | ENSMUSG00000061474 | -2,1 | 0,011 |
| Translation | **Mrpl12** | ENSMUSG00000039640 | -2,1 | 0,018 |
| Translation | **Mrps2** | ENSMUSG00000035772 | -2,1 | 0,018 |
| Translation | **C1qbp** | ENSMUSG00000018446 | -2,1 | 0,009 |
| Translation | **Ict1** | ENSMUSG00000018858 | -2 | 0,006 |
| Translation | **Vars2** | ENSMUSG00000038838 | -2 | 0,004 |
| Translation | **Mrpl47** | ENSMUSG00000037531 | -2 | 0,022 |
| Translation | **Mrpl21** | ENSMUSG00000024829 | -1,9 | 0,006 |
| Translation | **Mrps22** | ENSMUSG00000032459 | -1,9 | 0,008 |
| Translation | **Mrps16** | ENSMUSG00000049960 | -1,9 | 0,019 |
| Translation | **Mrpl34** | ENSMUSG00000034880 | -1,9 | 0,026 |
| Translation | **Mrpl23** | ENSMUSG00000037772 | -1,9 | 0,034 |
| Translation | **Trmu** | ENSMUSG00000022386 | -1,8 | 0,004 |
| Translation | **Tsfm** | ENSMUSG00000040521 | -1,8 | 0,014 |
| Translation | Trmt2b | ENSMUSG00000067369 | -1,8 | 0,021 |
| Translation | **Mrpl19** | ENSMUSG00000030045 | -1,8 | 0,027 |
| Translation | Mrpl38 | ENSMUSG00000020775 | -1,8 | 0,040 |
| Translation | **Pdf** | ENSMUSG00000078931 | -1,8 | 0,019 |
| Translation | Rpusd3 | ENSMUSG00000051169 | -1,8 | 0,035 |
| Translation | **Mrps5** | ENSMUSG00000027374 | -1,7 | 0,004 |
| Translation | Mtg2 | ENSMUSG00000039069 | -1,7 | 0,005 |
| Translation | **Mrpl18** | ENSMUSG00000057388 | -1,7 | 0,015 |
| Translation | **Mrpl15** | ENSMUSG00000033845 | -1,7 | 0,022 |
| Translation | **Mrpl24** | ENSMUSG00000019710 | -1,7 | 0,022 |
| Translation | **Mrps33** | ENSMUSG00000029918 | -1,7 | 0,041 |
| Translation | **Mars2** | ENSMUSG00000046994 | -1,7 | 0,015 |
| Translation | **Guf1** | ENSMUSG00000029208 | -1,7 | 0,026 |
| Translation | **Mrps17** | ENSMUSG00000034211 | -1,6 | 0,005 |
| Translation | **Mrps23** | ENSMUSG00000023723 | -1,6 | 0,030 |
| Translation | **Mrps18b** | ENSMUSG00000024436 | -1,6 | 0,033 |
| Translation | Thg1l | ENSMUSG00000011254 | -1,6 | 0,010 |
| Translation | Mtrf1l | ENSMUSG00000019774 | -1,5 | 0,007 |
| Translation | **Mtif2** | ENSMUSG00000020459 | -1,5 | 0,016 |
| Translation | **Yars2** | ENSMUSG00000022792 | -1,5 | 0,017 |
| Translation | **Mrpl4** | ENSMUSG00000003299 | -1,5 | 0,017 |
| Translation | Trmt10c | ENSMUSG00000044763 | -1,5 | 0,030 |
| Translation | **Mrpl46** | ENSMUSG00000030612 | -1,5 | 0,030 |
| Translation | **Ears2** | ENSMUSG00000030871 | -1,5 | 0,031 |
| Translation | **Mrpl22** | ENSMUSG00000020514 | -1,5 | 0,032 |
| Translation | **Mrpl28** | ENSMUSG00000024181 | -1,5 | 0,044 |
| Translation | Tars | ENSMUSG00000022241 | -1,4 | 0,009 |
| Translation | **Mrpl3** | ENSMUSG00000032563 | -1,4 | 0,015 |
| Translation | **Mrpl43** | ENSMUSG00000025208 | -1,4 | 0,020 |
| Translation | **Aars2** | ENSMUSG00000023938 | -1,4 | 0,026 |
| Translation | **Mrpl45** | ENSMUSG00000018882 | -1,4 | 0,032 |
| Translation | **Gars** | ENSMUSG00000029777 | -1,4 | 0,034 |
| Translation | **Kars** | ENSMUSG00000031948 | -1,4 | 0,047 |
| Translation | Rars | ENSMUSG00000018848 | -1,4 | 0,021 |
| Translation | Secisbp2 | ENSMUSG00000035139 | -1,4 | 0,027 |
| Translation | Ftsj2 | ENSMUSG00000029557 | -1,4 | 0,041 |
| Uncoupling | **Ucp1** | ENSMUSG00000031710 | -7,3 | 0,042 |
| Unknown | **Acyp2** | ENSMUSG00000060923 | -1,7 | 0,037 |
| Unknown | **Ccdc58** | ENSMUSG00000075229 | -1,5 | 0,024 |
| Various metabolism | **Gk** | ENSMUSG00000025059 | -3,1 | 0,004 |
| Various metabolism | Shmt1 | ENSMUSG00000020534 | -2,8 | 0,016 |
| Various metabolism | **Akr1b10** | ENSMUSG00000061758 | -2,2 | 0,025 |
| Various metabolism | **Carkd** | ENSMUSG00000031505 | -2,1 | 0,000 |
| Various metabolism | **Comtd1** | ENSMUSG00000021773 | -1,7 | 0,019 |
| Various metabolism | **Acad8** | ENSMUSG00000031969 | -1,4 | 0,019 |
| Various metabolism | **Pycr1** | ENSMUSG00000025140 | -9,2 | 0,000 |
| Various metabolism | Mthfd2 | ENSMUSG00000005667 | -4,8 | 0,001 |

Bold symbols are of genes which mitochondrial localization is proven for the white adipose tissue.

**Supplementary Table 4:** Functional categorization of genes upregulated at postnatal day 98 compared to 21, with strong support of mitochondrial localization. Expression of transcripts analyzed with mRNA sequence, of the transcripts with p<0.05 genes encoding proteins with strong support of mitochondrial localization (Mouse MitoCarta 2.0, Broad institute) were selected and functional categorized with Nextprot (SIB Swiss institute for bioinformatics)

| **Functional Category** | **Symbol** | **EnsemblGeneID** | **FC** | **P.Value** |
| --- | --- | --- | --- | --- |
| Apoptosis | Bcl2l2 | ENSMUSG00000089682 | 1,7 | 0,006 |
| Apoptosis | **Ghitm** | ENSMUSG00000041028 | 1,8 | 0,050 |
| Apoptosis | **Mtch1** | ENSMUSG00000024012 | 2,0 | 0,008 |
| Apoptosis | **Fhit** | ENSMUSG00000060579 | 3,3 | 0,004 |
| BCAA/SCFA metabolism | **Aldh6a1** | ENSMUSG00000021238 | 1,4 | 0,026 |
| BCAA/SCFA metabolism | **Pcca** | ENSMUSG00000041650 | 1,4 | 0,048 |
| BCAA/SCFA metabolism | **Prdx6** | ENSMUSG00000026701 | 1,5 | 0,029 |
| BCAA/SCFA metabolism | **Auh** | ENSMUSG00000021460 | 1,5 | 0,022 |
| BCAA/SCFA metabolism | **Ivd** | ENSMUSG00000027332 | 1,6 | 0,008 |
| BCAA/SCFA metabolism | **Lyplal1** | ENSMUSG00000039246 | 2,0 | 0,006 |
| BCAA/SCFA metabolism | **Gpt2** | ENSMUSG00000031700 | 2,1 | 0,003 |
| BCAA/SCFA metabolism | **Abat** | ENSMUSG00000057880 | 3,2 | 0,021 |
| BCAA/SCFA metabolism | Sugct | ENSMUSG00000055137 | 3,6 | 0,008 |
| BCAA/SCFA metabolism | **Mccc1** | ENSMUSG00000027709 | 33217 | 0,002 |
| Dynamics/Morphology | **Slc25a46** | ENSMUSG00000024259 | 1,9 | 0,001 |
| FA metabolism | **Acsl1** | ENSMUSG00000018796 | 1,8 | 0,044 |
| FA oxidation | **Echdc2** | ENSMUSG00000028601 | 2,1 | 0,002 |
| FA oxidation | **Acox1** | ENSMUSG00000020777 | 2,9 | 0,000 |
| FA oxidation | **Hsd17b4** | ENSMUSG00000024507 | 1,7 | 0,008 |
| FA oxidation | **Abcd2** | ENSMUSG00000055782 | 2,1 | 0,002 |
| FA oxidation | **Acad11** | ENSMUSG00000090150 | 3,3 | 0,001 |
| FA oxidation | **Pccb** | ENSMUSG00000032527 | 2,0 | 0,016 |
| FA synthesis | **Acss3** | ENSMUSG00000035948 | 5,2 | 0,000 |
| FA synthesis | **Acsm3** | ENSMUSG00000030935 | 5,3 | 0,032 |
| FA synthesis | **Acsm5** | ENSMUSG00000030972 | 5,3 | 0,000 |
| Fe/FeS | **Iscu** | ENSMUSG00000025825 | 1,5 | 0,011 |
| Fe/FeS | Sfxn4 | ENSMUSG00000063698 | 2,5 | 0,035 |
| Fe/FeS | **Bola1** | ENSMUSG00000015943 | 2,9 | 0,022 |
| Fe/FeS | Ndor1 | ENSMUSG00000006471 | 1,4 | 0,031 |
| Metabolic signalling | **Aldh1a7** | ENSMUSG00000024747 | 2,0 | 0,011 |
| Metabolic signalling | Ncoa4 | ENSMUSG00000056234 | 1,8 | 0,003 |
| Metabolite transport | **Slc25a26** | ENSMUSG00000045100 | 1,4 | 0,028 |
| Metabolite transport | Slc22a4 | ENSMUSG00000020334 | 2,4 | 0,001 |
| Mitochondrial replication | **Tk2** | ENSMUSG00000035824 | 1,5 | 0,034 |
| Mitophagy | Stx17 | ENSMUSG00000061455 | 1,8 | 0,003 |
| Mitophagy | Kif1b | ENSMUSG00000063077 | 2,3 | 0,002 |
| Mitophagy | Pink1 | ENSMUSG00000028756 | 2,7 | 0,000 |
| Mitophagy | **Bnip3** | ENSMUSG00000078566 | 2,7 | 0,000 |
| Mitophagy | Bnip3l | ENSMUSG00000022051 | 299,3 | 0,011 |
| Non mitochondrial | Dmpk | ENSMUSG00000030409 | 2,3 | 0,004 |
| Non mitochondrial | Ttc7b | ENSMUSG00000033530 | 1,7 | 0,007 |
| Non mitochondrial | **Tkt** | ENSMUSG00000021957 | 2,2 | 0,045 |
| Non mitochondrial | **Ephx2** | ENSMUSG00000022040 | 2,2 | 0,008 |
| Other | Prepl | ENSMUSG00000024127 | 1,5 | 0,044 |
| Other | **Pxmp2** | ENSMUSG00000029499 | 2,7 | 0,020 |
| Other | **Pxmp4** | ENSMUSG00000000876 | 1,4 | 0,046 |
| OXPHOS | **Atp5h** | ENSMUSG00000034566 | 1,5 | 0,025 |
| OXPHOS | **Surf1** | ENSMUSG00000015790 | 3,5 | 0,007 |
| OXPHOS | Cox19 | ENSMUSG00000045438 | 3,9 | 0,017 |
| Protein import and folding | **Clpb** | ENSMUSG00000001829 | 1,5 | 0,046 |
| Protein import and folding | Nrd1 | ENSMUSG00000053510 | 1,6 | 0,016 |
| Protein import and folding | **Tomm40l** | ENSMUSG00000005674 | 3,0 | 0,019 |
| Protein import and folding | **Chchd4** | ENSMUSG00000034203 | 2,8 | 0,000 |
| Redox | **Msra** | ENSMUSG00000054733 | 1,5 | 0,016 |
| Redox | **Gpx1** | ENSMUSG00000063856 | 1,7 | 0,029 |
| Redox | **Mgst1** | ENSMUSG00000008540 | 2,1 | 0,000 |
| Redox | **Cat** | ENSMUSG00000027187 | 2,2 | 0,001 |
| Redox | **Fam213a** | ENSMUSG00000021792 | 2,7 | 0,004 |
| Redox | **Htatip2** | ENSMUSG00000039745 | 2,7 | 0,000 |
| Redox | **Mgst3** | ENSMUSG00000026688 | 2,9 | 0,002 |
| Sterol metabolism | **Cyp27a1** | ENSMUSG00000026170 | 1,5 | 0,003 |
| Sterol metabolism | Serac1 | ENSMUSG00000015659 | 2,4 | 0,001 |
| Sterol metabolism | **Cyb5r3** | ENSMUSG00000018042 | 1,7 | 0,006 |
| Transcription | **Lrpprc** | ENSMUSG00000024120 | 224,9 | 0,009 |
| Translation | **Trnt1** | ENSMUSG00000013736 | 1,6 | 0,030 |
| Translation | Mtg1 | ENSMUSG00000039018 | 1,6 | 0,015 |
| Translation | **Mrps6** | ENSMUSG00000039680 | 1,7 | 0,038 |
| Translation | **Mrpl41** | ENSMUSG00000036850 | 1,8 | 0,014 |
| Translation | **Hrsp12** | ENSMUSG00000022323 | 3,7 | 0,002 |
| Unknown | **Tmem205** | ENSMUSG00000040883 | 308,5 | 0,010 |
| Various metabolism | Asah2 | ENSMUSG00000024887 | 1,5 | 0,028 |
| Various metabolism | **Mdh1** | ENSMUSG00000020321 | 1,6 | 0,013 |
| Various metabolism | **Pdp1** | ENSMUSG00000049225 | 5,3 | 0,022 |
| Various metabolism | **Dhrs7b** | ENSMUSG00000042569 | 1,5 | 0,045 |
| Various metabolism | **Sqrdl** | ENSMUSG00000005803 | 1,6 | 0,012 |
| Various metabolism | **Maob** | ENSMUSG00000040147 | 1,7 | 0,023 |
| Various metabolism | **Dglucy** | ENSMUSG00000021185 | 1,7 | 0,008 |
| Various metabolism | **Acad12** | ENSMUSG00000042647 | 1,9 | 0,018 |
| Various metabolism | **Maoa** | ENSMUSG00000025037 | 2,0 | 0,002 |
| Various metabolism | **Car5b** | ENSMUSG00000031373 | 2,0 | 0,027 |
| Various metabolism | **Fahd2a** | ENSMUSG00000027371 | 2,1 | 0,011 |
| Various metabolism | **Cyb5** | ENSMUSG00000024646 | 2,2 | 0,002 |
| Various metabolism | Sdsl | ENSMUSG00000029596 | 2,2 | 0,006 |
| Various metabolism | Chpt1 | ENSMUSG00000060002 | 2,3 | 0,005 |
| Various metabolism | **Aldh2** | ENSMUSG00000029455 | 2,4 | 0,001 |
| Various metabolism | **Ldhd** | ENSMUSG00000031958 | 2,6 | 0,015 |
| Various metabolism | **Gldc** | ENSMUSG00000024827 | 4,5 | 0,000 |
| Various metabolism | **Crat** | ENSMUSG00000026853 | 1,9 | 0,027 |

Bold symbols are of genes which mitochondrial localization is proven for the white adipose tissue.

1. Meng L, Jan SZ, Hamer G, van Pelt AM, van der Stelt I, Keijer J, et al. Preantral follicular atresia occurs mainly through autophagy, while antral follicles degenerate mostly through apoptosis. *Biol Reprod* (2018) 99(4):853-63. doi: 10.1093/biolre/ioy116. PubMed PMID: 29767707.

2. Gesta S, Tseng YH, Kahn CR. Developmental origin of fat: tracking obesity to its source. *Cell* (2007) 131(2):242-56. doi: 10.1016/j.cell.2007.10.004. PubMed PMID: 17956727.
