# Supplementary figures and images for: Maturation of White Adipose Tissue Function in C57BL/6j Mice From Weaning to Young Adulthood
Source: Front Physiol. 2019 Jul 9;10:836. doi: 10.3389/fphys.2019.00836 (PMC6629938; doi:10.3389/fphys.2019.00836)

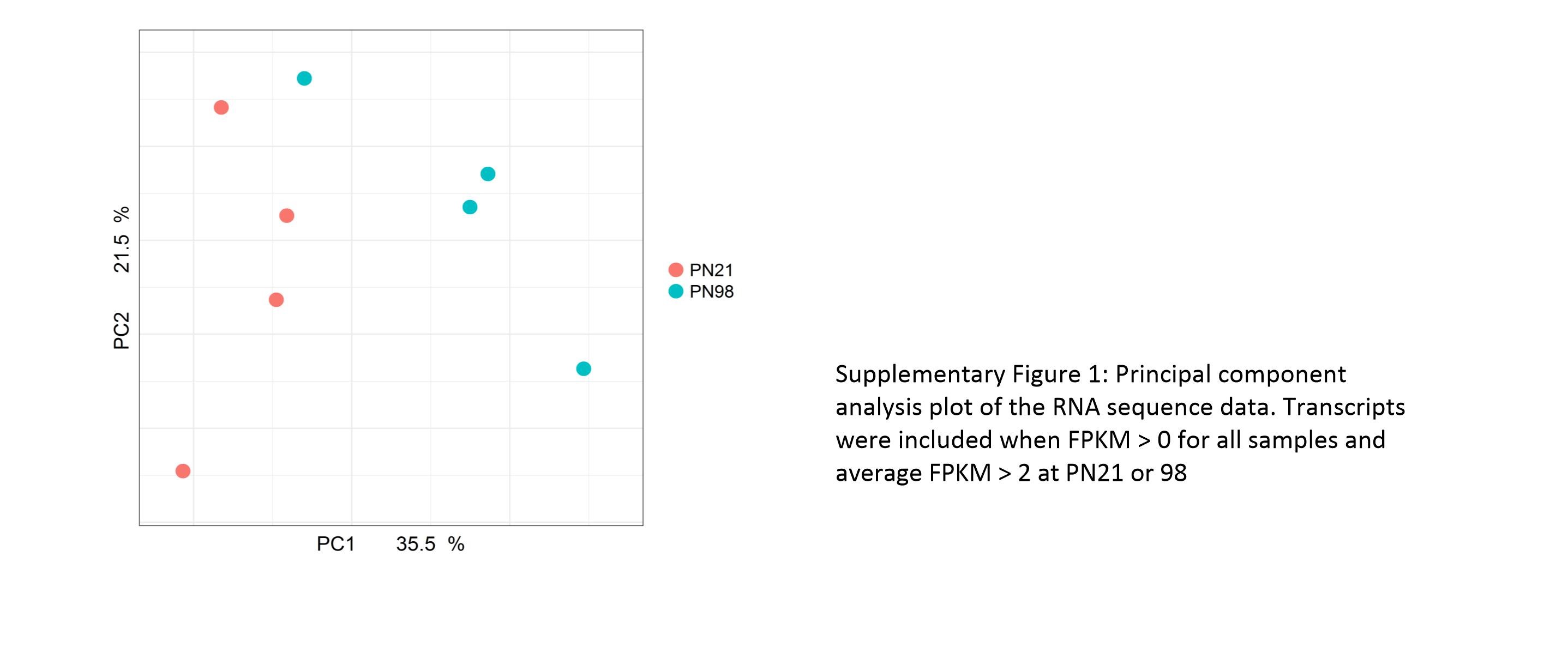

Supplement: Supplementary file 2 [file Image_1.jpeg]

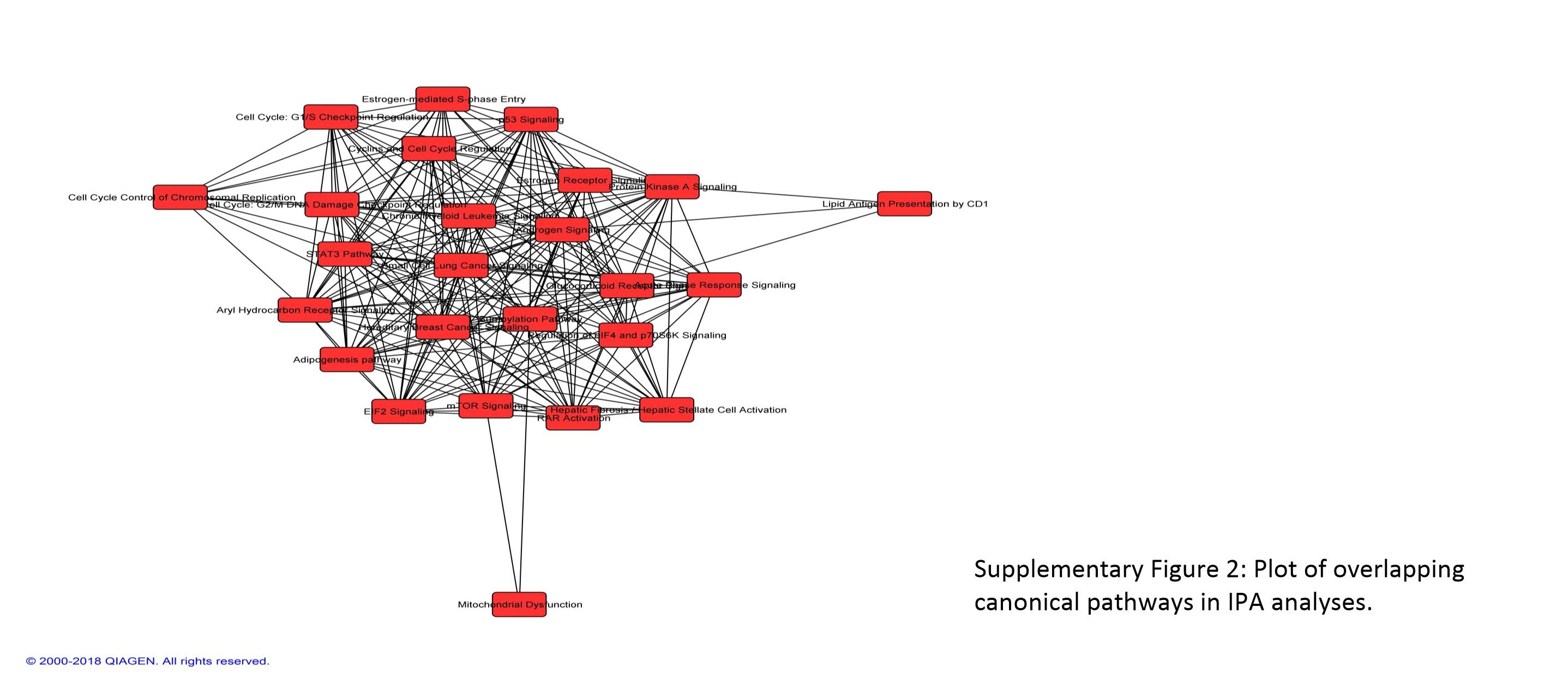

Supplement: Supplementary file 3 [file Image_2.jpeg]

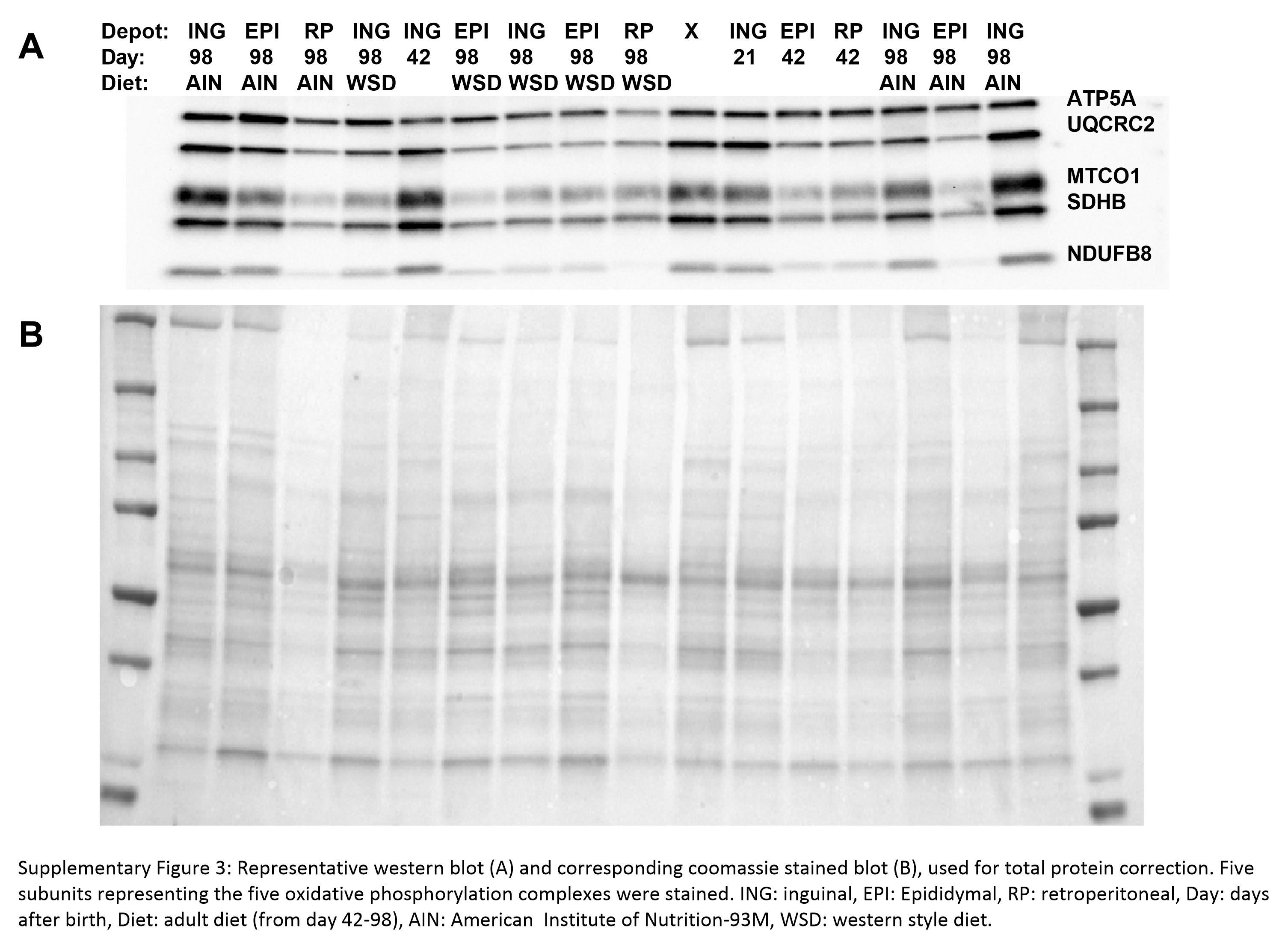

Supplement: Supplementary file 4 [file Image_3.jpeg]

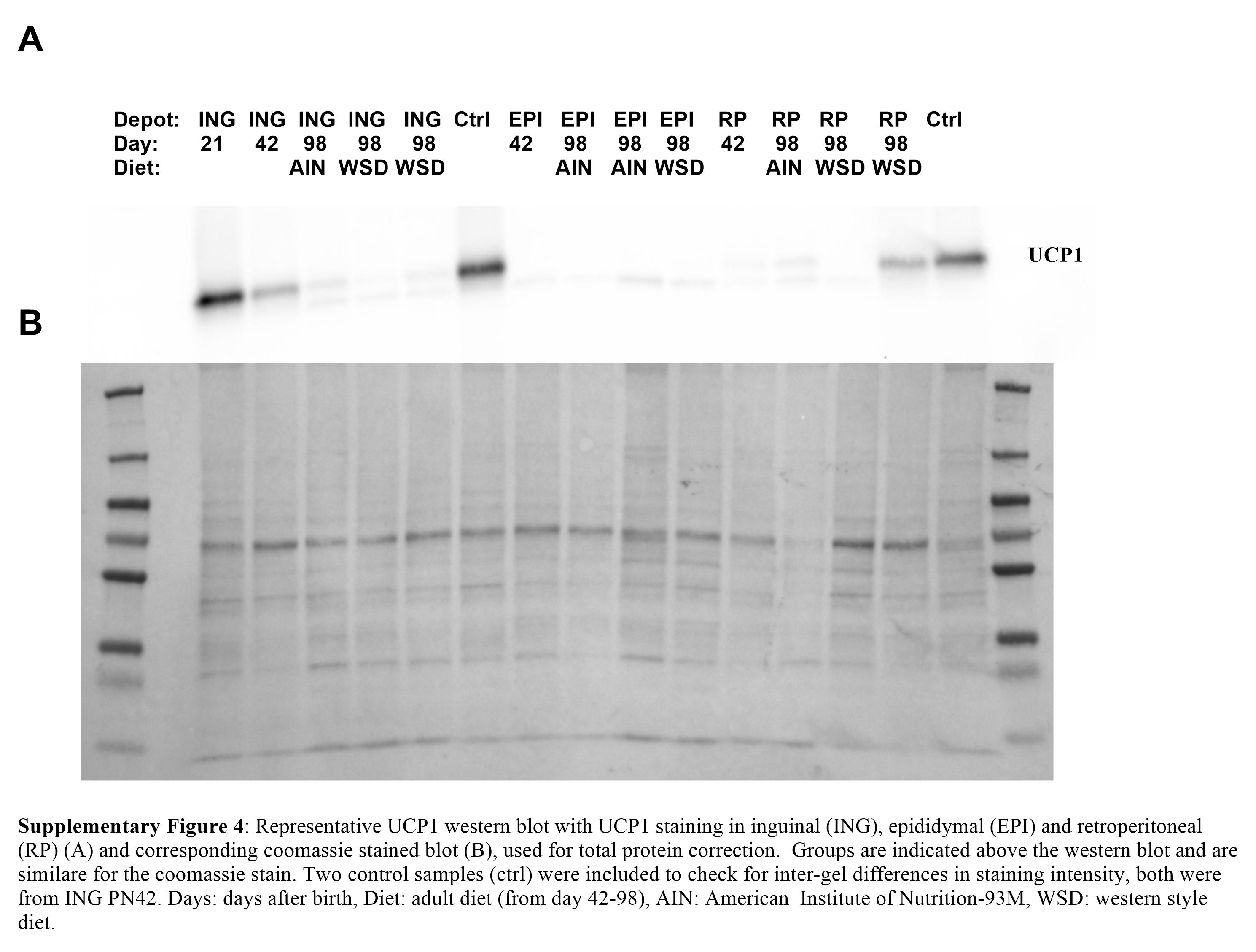

Supplement: Supplementary file 5 [file Image_4.jpeg]

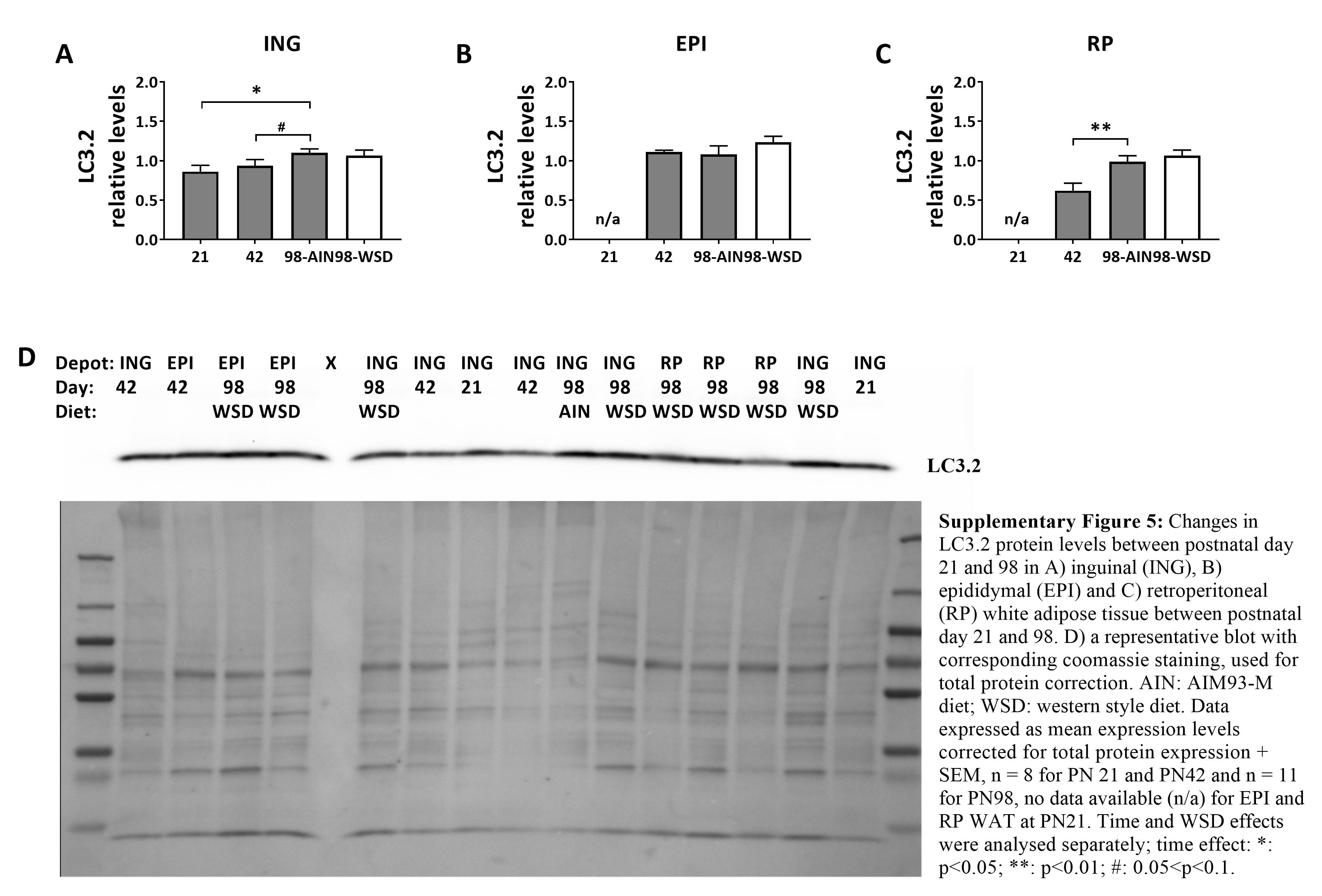

Supplement: Supplementary file 6 [file Image_5.jpeg]
